# Zika virus enhances monocyte adhesion and transmigration favoring viral dissemination to neural cells

**DOI:** 10.1038/s41467-019-12408-x

**Published:** 2019-09-27

**Authors:** Nilda Vanesa Ayala-Nunez, Gautier Follain, François Delalande, Aurélie Hirschler, Emma Partiot, Gillian L. Hale, Brigid C. Bollweg, Judith Roels, Maxime Chazal, Florian Bakoa, Margot Carocci, Sandrine Bourdoulous, Orestis Faklaris, Sherif R. Zaki, Anita Eckly, Béatrice Uring-Lambert, Frédéric Doussau, Sarah Cianferani, Christine Carapito, Frank M. J. Jacobs, Nolwenn Jouvenet, Jacky G. Goetz, Raphael Gaudin

**Affiliations:** 1 Institut de Recherche en Infectiologie de Montpellier (IRIM), CNRS, Université de Montpellier, 34293 Montpellier, France; 2 Université de Strasbourg, INSERM, 67000 Strasbourg, France; 3 INSERM U1109 and FMTS, 67000 Strasbourg, France; 4 Laboratoire de Spectrométrie de Masse Bio-Organique, IPHC, UMR 7178, CNRS-Université de Strasbourg, ECPM, 67087 Strasbourg, France; 5 Infectious Diseases Pathology Branch, Division of High-Consequence Pathogens and Pathology, National Center for Emerging and Zoonotic Infectious Diseases (NCEZID), Centers for Disease Control and Prevention, 1600 Clifton Rd NE, MS: G32, Atlanta, GA 30329-4027 USA; 6 University of Amsterdam, Swammerdam Institute for Life Sciences, Science Park 904, 1098XH Amsterdam, The Netherlands; 7 Viral Genomics and Vaccination Unit, UMR3569 CNRS, Virology Department, Institut Pasteur, 75015 Paris, France; 8 Université de Strasbourg, INSERM, EFS Grand Est, BPPS UMR-S1255, FMTS, 67000 Strasbourg, France; 9 INSERM U1016, Institut Cochin, CNRS UMR8104, Université Paris Descartes, Paris, France; 10 MRI Core facility, Biocampus, CNRS UMS 3426, 34293 Montpellier, France; 11 Hôpitaux universitaires de Strasbourg, laboratoire central d’immunologie, 67000 Strasbourg, France; 12 Institut des Neurosciences Cellulaires et Intégratives, CNRS, Université de Strasbourg, 67000 Strasbourg, France

**Keywords:** Zebrafish, Cell adhesion, Mechanisms of disease, Cellular microbiology, Virology

## Abstract

Zika virus (ZIKV) invades and persists in the central nervous system (CNS), causing severe neurological diseases. However the virus journey, from the bloodstream to tissues through a mature endothelium, remains unclear. Here, we show that ZIKV-infected monocytes represent suitable carriers for viral dissemination to the CNS using human primary monocytes, cerebral organoids derived from embryonic stem cells, organotypic mouse cerebellar slices, a xenotypic human-zebrafish model, and human fetus brain samples. We find that ZIKV-exposed monocytes exhibit higher expression of adhesion molecules, and higher abilities to attach onto the vessel wall and transmigrate across endothelia. This phenotype is associated to enhanced monocyte-mediated ZIKV dissemination to neural cells. Together, our data show that ZIKV manipulates the monocyte adhesive properties and enhances monocyte transmigration and viral dissemination to neural cells. Monocyte transmigration may represent an important mechanism required for viral tissue invasion and persistence that could be specifically targeted for therapeutic intervention.

## Abstract (web summary)

Zika virus (ZIKV) can infect the central nervous system, but it is not clear how it reaches the brain. Here, Ayala-Nunez et al. show in ex vivo and in vivo models that ZIKV can hitch a ride in monocytes in a Trojan Horse manner to cross the endothelium and disseminate the virus.

## Introduction

Zika virus (ZIKV) is a major public health concern worldwide^1^. Currently, no approved vaccine or drugs are available for treatment. The virus is a blood-borne pathogen from the *Flaviviridae* family that is transmitted through the bite of an infected mosquito but also by human-to-human sexual transmission, blood transfusion, and mother-to-child transfer during pregnancy or at delivery. The most severe complications include fetal microcephaly in pregnant women, Guillain–Barré syndrome, as well as other neurological disorders not only in fetuses, but also in newborns, infants, and adults, severe thrombocytopenia, and testicular damage and atrophy^1–5^. The wide dissemination of the virus within the body suggests that molecular and cellular mechanisms from the host are subverted to allow ZIKV virions to travel from their port of entry toward tissues. This is particularly important for the difficult-to-access brain sanctuary. ZIKV efficiently invades and persists within the brain^6–8^ and exhibits a preferential tropism for human neural progenitor cells (hNPCs), which are key players in the development of ZIKV-induced neurological diseases^2,9–11^. However, the mechanism by which ZIKV travels toward and spreads into the brain remains unknown.

Although endothelial blood-to-tissue permeability may allow diffusive virus spreading in a first-trimester fetus, it is not clear how ZIKV would invade hard-to-reach tissues exhibiting a mature, impermeable endothelium. Yet, ZIKV efficiently reaches and remains within the brain of hosts with a mature blood–brain barrier (BBB)^6,7,12–14^. The BBB is an extremely tight endothelium separating bloodstream-circulating virions from the neural target cells. The Trojan Horse strategy, consisting of the infection of circulating leukocytes that carry virus through endothelial monolayers, has been proposed for numerous viruses in various in vitro infection assays^15–19^, but never highlighted in an in vivo context. Monocytes are considered as well-suited viral carriers since they exhibit potent transmigrating abilities over endothelial barriers, including the BBB^20^. It was recently shown that circulating monocytes harbor ZIKV in vitro and in patients^21–23^, but no further role was attributed to these cells in the physiopathology of the infection.

Here, we show that ZIKV-infected monocyte-derived cells are found in the CNS of a human fetus with microcephaly and we assessed monocyte-driven ZIKV dissemination and damage in ex vivo culture models, including human embryonic stem cell (hESC)-derived cerebral organoids and organotypic mouse cerebellar slices. Moreover, we find that exposure of human monocytes to ZIKV triggers higher expression of adhesion molecules, higher capacities to spread and adhere to different substrates, and higher abilities to attach and transmigrate through endothelia in vitro and in a zebrafish embryo model as compared with noninfected monocytes. Finally, we correlate the increased transmigration phenotype to higher dissemination rates to hESC-derived cerebral organoids compared with cell-free virus infection.

## Results

### ZIKV-infected monocyte-derived cells found in a human fetus CNS

**Fig. 1:**
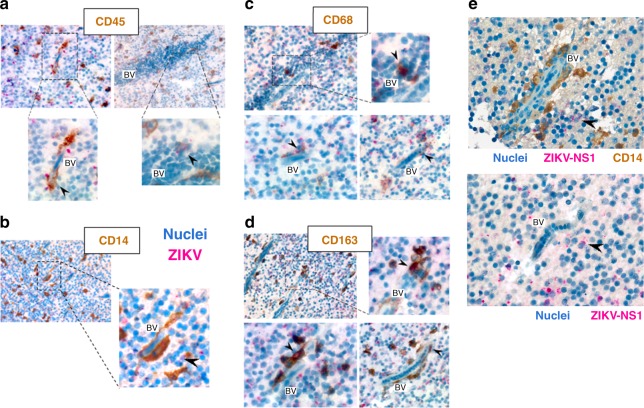
Monocyte-derived cells are infected by ZIKV in a human fetus with microcephaly. **a**–**e** Immunohistochemical staining was performed on human fetal brain tissues from a PCR-confirmed case of congenital ZIKV (gestational age 22 weeks). All slides were counterstained in Mayer’s Hematoxylin and blued in Lithium carbonate. The tissue slices were stained for ZIKV–NS1 in combination with **a** CD45 (left panel: 63×, right panel: 40×), **b** CD14 (20×), **c** CD68 (upper panel: 63×, lower left panel: 100×, and lower right panel: 40×), or **d** CD163 (upper panel: 40×, lower left panel: 100×, and lower right panel: 63×). **e** The endothelial cells of slices from the same tissue appear morphologically intact and were never found positive for ZIKV–NS1 staining. The black arrowheads indicate ZIKV-infected cells. BV blood vessel

First, we asked whether ZIKV-infected monocyte-derived cells could be detected in human brain samples. Brain slices of a ZIKV-positive human fetus (5 months) diagnosed with microcephaly were stained for the viral protein NS1 together with the leukocyte marker CD45, the monocytic marker CD14, or the myeloid markers CD68 or CD163. Numerous cells expressing these markers in the perivascular area were found positive for ZIKV–NS1 (Fig. 1a–d and controls in Supplementary Fig. 1). Importantly, although endothelial cells have been reported to be targets of ZIKV in vitro^24–26^, we did not observe any infection of these cells from the BBB of a naturally ZIKV-infected human fetus with microcephaly (Fig. 1e).

### Monocytes are productively infected by ZIKV

To assess for the susceptibility of leukocytes to ZIKV infection, we isolated total PBMCs from three human healthy donors infected in vitro by ZIKV strains isolated in 2015 in Colombia (ZIKV^C^) and Panama (ZIKV^P^) that share 99.5% similarity. Flow cytometry analysis was performed to define the permissiveness of CD4^+^ T lymphocytes, CD8^+^ T lymphocytes, B lymphocytes, monocytes, natural killer (NK) cells, and NKT cells (Supplementary Fig. 2a). A primary labeled anti-NS2B antibody that recognizes a nonstructural ZIKV protein (that is not contained into incoming virions) was used to identify cells supporting active ZIKV replication (as opposed to noninfected cells carrying incoming particles). All cell types showed some level of ZIKV infection, in a strain- and donor-dependent manner (Supplementary Fig. 2a). Monocytes were the most permissive cell type overall, in agreement with previous reports^21–23^. Moreover, ZIKV induced a shift of the monocyte population from a CD14^+^ CD16^−^ toward a CD14^+^ CD16^+^, preferentially infecting them (Supplementary Fig. 2b, c), which correlates with studies that previously showed that the CD14^+^ CD16^+^ monocytic subset is particularly permissive to infection by other Asian ZIKV strains^21,22^.

**Fig. 2:**
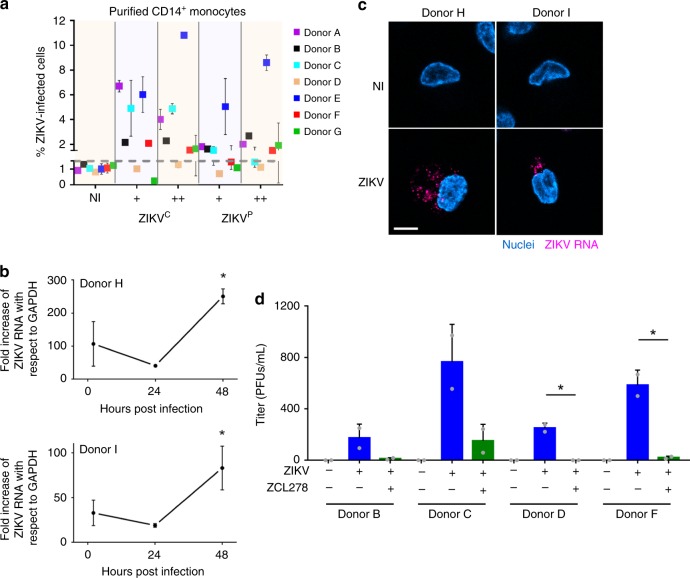
Human primary monocytes are productively infected by ZIKV. **a** Monocytes purified from seven healthy blood donors were infected with Colombian (ZIKV^C^) or Panama (ZIKV^P^) ZIKV strains at MOI 1 (+) or 5 (++) for 48 h. Cells were fixed, permeabilized, and processed for flow cytometry. The percentage of ZIKV-infected cells (NS2B^+^) is plotted as a function of the strain and amount of ZIKV used. Each dot corresponds to the mean ± SD of ZIKV-infected cells for each donor and each condition in two individual samples. **b** Monocytes were infected for the indicated time with ZIKV (MOI 1) for 48 h. Total RNA was extracted and RT-qPCR was performed by using ZIKV and GAPDH-specific primers. The graph represents the fold change of ZIKV RNA with respect to GAPDH of a triplicate ± SD. Data from two donors are shown. No ZIKV RNA was detected in monocytes from a third donor in this experiment (data provided in the Source Data file). **c** Monocytes infected with ZIKV for 48 h were fixed and fluorescence in situ hybridization (FISH) was performed by using a ZIKV-specific RNA probe (magenta) and nuclei were stained with NucBlue (blue). Left and right panels are representative images from experiments done with two donors. Scale bar: 5 µm. **d** Monocytes purified from four healthy donors were untreated or pretreated with 50 µM ZCL278 for 30 min at 37 °C. The cells were then infected with ZIKV (MOI 1) in the presence or absence of ZCL278. The inoculum was removed at 4 h post infection, after which the cells were washed and re-incubated in complete media for 48 h. The supernatant was harvested and a plaque assay on Vero cells was performed to titrate ZIKV production. The bar graphs represent the mean + SD of two independent samples. Each dot represents the mean of a duplicate. ZCL278 treatment inhibited ZIKV infection of monocytes by 93% (±7). The letters attributed to each donor correlate with the ones in **a**. The two-tailed *p* value was <0.05 (*). Statistical significance was determined by using a *t* test. NI noninfected. Source data in **a**–**d** are provided as a Source Data file

Given the variability observed among the three blood donors examined, we then purified monocytes from PBMCs of these three donors (donors A–C) as well as from four additional donors (donors D–G). Flow cytometry analyses revealed that monocytes were permissive to ZIKV^C^ in six out of seven donors and to ZIKV^P^ in five out of seven donors (Fig. 2a). In the subsequent experiments, we worked solely with the ZIKV^C^ strain, which shows better infectiveness, and will be referred to as ZIKV.

To determine whether the absence of NS2B staining in some donors was due to low fluorescence sensitivity, we performed reverse transcription quantitative polymerase chain reaction (RT-qPCR) analysis on monocytes at different times post infection. Successful replication was observed in two donors (Fig. 2b). Specifically, the amount of viral RNA was first decreasing from 2 to 24 hpi, indicative of the expected incoming viral RNA degradation, followed by an increase at 48 hpi. Such increase represents viral replication. Thus, ZIKV replication in monocytes is slow as compared with the 24-h cell cycle usually reported for Flaviviruses in other cell types (see for instance ref. ^27^). This late timing was also observed at the protein level, where NS2B was successfully detected at 48 but not at 24 hpi (Supplementary Fig. 3a). Detection of ZIKV RNA by fluorescence in situ hybridization (FISH) in monocytes isolated from two donors further demonstrated the ability of the virus to replicate in this cell type (Fig. 2c). In agreement with flow cytometry and qPCR data, very few cells were positive for viral RNA, suggesting that only a small subset of monocytes is susceptible to infection. Finally, productive monocyte infection was established by measuring infectious viral particle release and we found that monocytes shed a low amount of infectious ZIKV (Fig. 2d). ZCL278, a Cdc42 inhibitor^28^ and a broad-spectrum virus entry inhibitor^29^ abolished ZIKV infection of monocytes (Fig. 2d and Supplementary Fig. 3b). In all the conditions tested, we measured the amount of LDH released from dying cells as an indirect measurement of cytotoxicity and found that neither ZIKV nor ZCL278 induced cell death (Supplementary Fig. 3c).

### ZIKV-infected monocytes disseminate the infection

**Fig. 3:**
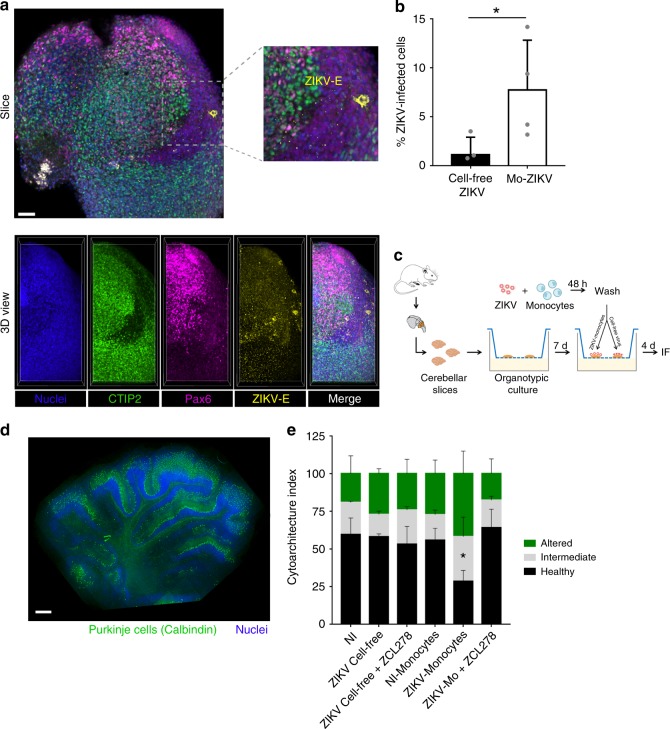
ZIKV-infected monocytes promote viral dissemination and tissue damage to cerebral organoids. **a**, **b** Cerebral organoids of 3 or 5 weeks post differentiation (wpd) were cocultured with ZIKV-infected monocytes, noninfected monocytes, cell-free ZIKV, or mock for 2 days. The organoids were then fixed and processed for **a** immunofluorescence or **b** flow cytometry. **a** A slice and the 3D view of the same organoid are shown, which was cocultured with ZIKV-infected monocytes. The crop shows infected cells (not monocytes) in yellow. Scale bar: 50 µm. **b** The bar graphs represent the mean of an experiment performed in duplicate ± SD with monocytes from two donors. **c**–**e** Organotypic cultures of mouse cerebellar slices were cultured in the presence or absence of cell-free ZIKV or ZIKV-infected monocytes ± ZCL278. **c** Experimental design. **d** A staining with an anti-calbindin antibody (green) and Dapi (blue) was done to observe the tissue cytoarchitecture. Scale bar: 500 µm. **e** The cytoarchitecture index is a measurement of the degree of tissue injury. Three categories were defined: healthy (part of the lobule with a regular alignment of Purkinje), intermediate (partially damaged, sparse Purkinje), or altered (absence of Purkinje, impaired on the slice). For each condition, the length of the healthy, intermediate, or altered regions was measured by using ImageJ and the proportion of each category was plotted. The condition in which cerebellar slices were incubated with ZIKV-infected monocytes is the only one that significantly differed from the others. Two-tailed *p* value < 0.05 (*). Statistical significance was determined by using a *t* test. NI noninfected. Source data in **b**, **e** are provided as a Source Data file

As monocytes are productively infected (Fig. 2), we then addressed whether they could disseminate ZIKV to target cells. Therefore, we grew cerebral organoids from hESCs^30,31^ to use them as a 3D model of neurological tissue (see Supplementary Fig. 4a and Materials and Methods for details). At 3 weeks post differentiation (wpd), the cells composing the cerebral organoids were mostly Pax6-positive, indicative of neural precursor cells (Supplementary Fig. 4b). At 5 wpd, the organoids contained Pax6-positive cells organized as Rosetta and a high number of terminally differentiated neurons, as shown by the increased number of CTIP2-positive cells (Supplementary Fig. 4c). The hESC-derived cerebral organoids were cocultured with cell-free ZIKV or ZIKV-infected monocytes for 2 days, and analyzed by flow cytometry to measure the percentage of live ZIKV-infected cells among the CCR2-negative population (to exclude the monocytes). Strikingly, we observed that ZIKV-infected monocytes isolated from two donors were successful at disseminating the infection to the organoids as early as 2 days post contact, while the ability of cell-free virus to disseminate the infection was marginal to null (Fig. 3a, b and Supplementary Fig. 4d). When the infection was allowed for 9 days, the organoids infected with cell-free virus were successfully infected as well (Supplementary Fig. 4e–f), suggesting that monocytes accelerate the kinetics of ZIKV dissemination to cerebral organoids.

### ZIKV-infected monocytes alter the cytoarchitecture of the cerebellum

To further explore the functional relevance of the presence of infected monocytes in the CNS, we used an organotypic culture of mouse cerebellar slices^32^, a method of choice for the ex vivo study of viral infection. ZIKV was shown to induce abnormal cerebellum anatomy in mice^33^, and thus organotypic culture provides a tissue that maintains cellular composition, tissue architecture, and function of a bona fide cerebellum^32^. The cerebellar slices were incubated for 4 days with noninfected human monocytes, ZIKV-infected monocytes, or cell-free virus in the presence or absence of ZCL278 (scheme in Fig. 3c). Staining of the Purkinje cell layer and nuclei showed relatively well-conserved tissue architecture in untreated conditions (Fig. 3d). Quantification of the proportion of altered, intermediate, or healthy lobules highlighted that the cerebellar slices incubated with ZIKV-infected monocytes represent the only condition in which altered cytoarchitecture of the cerebellar lobules was significantly increased (Fig. 3e), suggesting that cell-associated viral dissemination favors neuropathogenesis. This phenotype was completely reversed by the use of the antiviral ZCL278.

### ZIKV-infected monocytes exhibit higher transmigration abilities

**Fig. 4:**
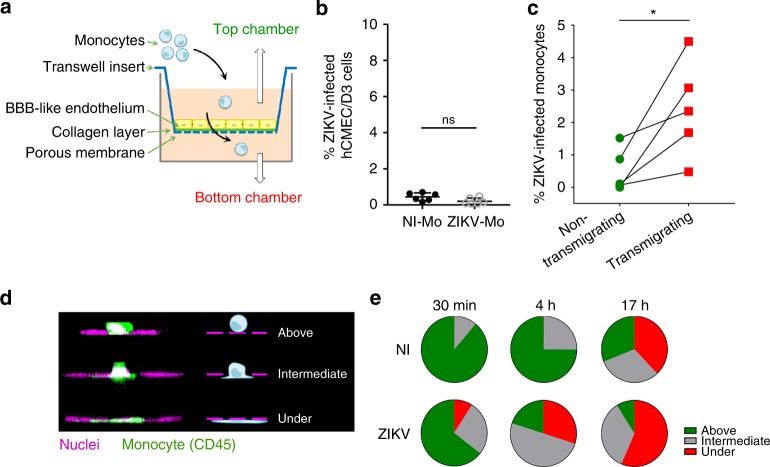
ZIKV-infected monocytes exhibit higher transmigration properties. **a** Representative scheme of the transwell transmigration assay. **b** After a transmigration assay was performed, the hCMEC/D3 monolayer was harvested from the transwell membrane, fixed, and processed for flow cytometry. The cells were stained with a NS2B antibody to assess for viral infection. The graph shows the mean ± SD of two independent experiments done in triplicate with two donors. **c** Monocytes were noninfected or infected with ZIKV at MOI 1 for 48 h. Upon extensive washes, monocytes were added on top of a transwell insert onto which hCMEC/D3 endothelial cells were plated 7 days before addition. Transmigration was allowed to occur for ≈17 h. Monocytes from the top and bottom chambers were harvested, fixed, and stained. Flow cytometry analysis was performed to determine the percentage of ZIKV-infected cells (NS2B^+^) among the monocytes that did not transmigrate (top, green circles) or did transmigrate (bottom, red squares). The graph shows two individual experiments from three donors. One of the replicates was not presented, because no infected cells were detected in the top or bottom chambers (see Source Data file for details). Monocytes from the top and bottom come from the same transwell. The fold change of the percentage of infected cells in the top and bottom chambers was significantly greater (two-way Anova = 0.039). **d**–**e** ZIKV-infected or noninfected (NI) monocytes isolated from two donors were cocultured for 17 h with a hCMEC/D3 monolayer previously grown on glass coverslips for 7 days. Upon fixation and staining with a fluorescently labeled anti-CD45 antibody, the number of monocytes located above, intermediate, or under the endothelial layer (as depicted in **d**) was quantified by confocal microscopy. **e** The pie charts correspond to the number of monocytes counted per ten fields of view from two individual experiments with two donors. Two-tailed *p* value < 0.05 (*). Statistical significance was determined by using a *t* test. NI noninfected, ns nonsignificant. Source data in **b**, **c**, **e** are provided as a Source Data file

To test whether ZIKV could influence circulating monocytes to cross the BBB, transwell inserts were seeded with the human cerebellar microvascular endothelial cell D3 (hCMEC/D3)^34^ (see scheme Fig. 4a). Under our culturing conditions, hCMEC/D3 cells form a tight monolayer exhibiting robust impermeability (Supplementary Fig. 5a–b), within the range of expected tightness^35^. ZIKV-infected monocytes, cell-free virus, and ZCL278 treatment did not significantly perturb endothelial impermeability (Supplementary Fig. 5c). No viral replication was observed in the hCMEC/D3 monolayer during the course of the experiment (Fig. 4b). However, a significantly greater percentage of monocytes that transmigrated were ZIKV-positive (bottom chamber, green) compared with the one that did not transmigrate (top chamber, red; Fig. 4c). Consistently, scanning confocal microscopy analysis revealed that a greater proportion of monocytes were found under the endothelial monolayer when exposed to ZIKV (Fig. 4d, e).

Together, our data indicate that ZIKV readily enhances the ability of monocytes to transmigrate, while the permeability of the endothelial layer remains unaffected.

### ZIKV-exposed monocytes exhibit higher adhesion properties

**Fig. 5:**
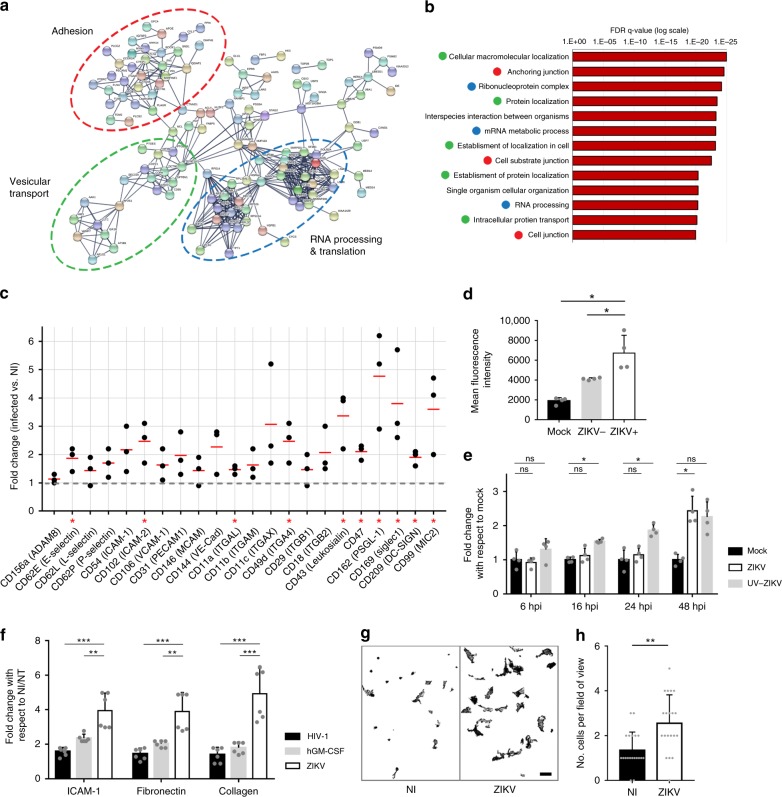
ZIKV-exposed monocytes exhibit higher adhesion properties. **a**, **b** Monocytes from two donors were infected with ZIKV. At 48 hpi, the cells were processed for quantitative proteome profiling by using liquid chromatography coupled to tandem mass spectrometry (LC–MS/MS). Cluster and ontology analyses of the upregulated proteins (virus over mock) were identified by using STRING (**a**) and GSEA (**b**) methods. The list of the proteins modulated upon ZIKV infection by using a 5% FDR (*p* value < 0.0085) is provided in Supplementary Data 1. **c** Flow cytometry analysis assessing the surface expression of 22 adhesion molecules upon ZIKV infection. Each dot corresponds to the average fold change of duplicates for individual donors. The red lines correspond to the mean from three donors. The gray dashed line indicates a fold change of 1 (no differential expression between infected and noninfected monocytes). **d** The expression of CD99 (MIC2) was measured in monocytes from two donors treated with mock or ZIKV for 48 h. In the latter, the CD99 expression was quantified within the noninfected (ZIKV–) and the infected (ZIKV+) populations. **e** Expression of CD99 was measured over time on monocytes from two donors treated with mock, ZIKV, and UV-inactivated ZIKV. The bar graphs show the fold change of CD99 expression with respect to mock (mean ± SD). **f** Noninfected/nontreated (NI/NT), hGM-CSF-treated, ZIKV-, or HIV-infected monocytes were plated in wells coated with collagen, ICAM-1 protein, or fibronectin. The relative number of cells was measured by using CellTiter-Glo. Each bar graph corresponds to an experiment performed on monocytes from two healthy donors showing the mean ± SD from two individual experiments. **g**, **h** ZIKV-infected or NI monocytes of two donors were cocultured with hCMEC/D3 cells grown on glass coverslips. Upon fixation and staining with an anti-CD45 antibody, the number of adherent monocytes was quantified by confocal microscopy (**g** and Supplementary Fig. 9a, b). Scale bar: 20 µm. **h** The bar graph corresponds to the mean ± SD of the number of monocytes counted per ten fields of view from two individual experiments for two donors. Two-tailed *p* value was nonsignificant (ns), <0.05 (*), <0.005 (**), or <0.0005 (***). Statistical significance was determined by using a *t* test. NI noninfected, NT no cytokine treatment. Source data in **b**–**f**, **h** are provided as a Source Data file

To further investigate the impact of ZIKV on human primary monocytes, we performed differential quantitative proteome profiling by using highly sensitive and robust liquid chromatography coupled to tandem mass spectrometry (LC–MS/MS). A total of 545 proteins were differentially expressed (198 upregulated and 347 downregulated) between mock and ZIKV-exposed monocytes from two donors (in quadruplicates, FDR 0.05; see Supplementary Data 1). Upon parallel analyses by using STRING^36^ (highest confidence 0.9) and GSEA^37^ (“biological process” and “cellular component” pathways), we observed that ZIKV induced a significant enrichment of proteins associated with adhesion, vesicular transport (clathrin-mediated endocytosis and cytoskeleton-related proteins), and RNA processing and translation (Fig. 5a, b). Specifically, integrins (β1, α5, and αM), ICAM3, PECAM1, IQGAP1, catenin, myosins, actinin, KIF5B, vinculin, talin, and filamin A and B that are involved in cell adhesion and/or in the establishment of focal adhesion sites, were upregulated by ZIKV (Supplementary Data 1). Proteins associated with the immune system response were not significantly upregulated in monocytes upon ZIKV infection. By comparison, monocytes exposed to HIV-1, which is unrelated to flaviviruses, or to the closely related Dengue 2 virus (DENV2), elicited an enrichment of proteins involved in antiviral immune response, but no modulation of adhesion-related proteins (Supplementary Fig. 6). Thus, the increase in expression of adhesion proteins in monocytes seems specific for ZIKV infection.

To determine whether the ZIKV-induced expression of adhesion molecules was overrepresented at their functional location, i.e., the cell surface, we next assessed by flow cytometry the monocyte’s surface expression levels of a panel of well-known adhesion molecules. We found that 10 out of the 22 proteins tested were significantly upregulated at the surface of monocytes from three donors (Fig. 5c). Interestingly, productive infection was not strictly required as noninfected bystander cells (Fig. 5d), and UV-inactivated ZIKV (Fig. 5e and Supplementary Fig. 7) also significantly induced the overexpression of the adhesion-associated protein CD99. Of note, UV-inactivated virus exposure induced an early overexpression of CD99 at the surface of monocytes (Fig. 5e) and therefore, the molecular mechanism behind this increase could differ from the one observed in ZIKV-infected monocytes. Interestingly, while CD99 and ITGAL were significantly overexpressed at the cell surface of monocytes (Fig. 5c–e), we observed a decreased expression of the mRNA coding for these proteins (Supplementary Fig. 8). As ZIKV enhances RNA processing and translation-related proteins (Fig. 5a, b), one could hypothesize that ZIKV lowers transcription levels and favors translation by an unknown mechanism.

We next investigated whether the observed ZIKV-induced adhesion phenotype would have a functional role on monocyte’s attachment. Upon ZIKV infection, adhesion of monocytes was significantly increased on different substrates and on hCMEC/D3 endothelial cells (Fig. 5f–h). This increased attachment was specific for ZIKV, since infection with HIV-1 and treatment with hGM-CSF did not result in such an increase (Fig. 5f).

### ZIKV-exposed monocytes exhibit increased spread-out morphology

**Fig. 6:**
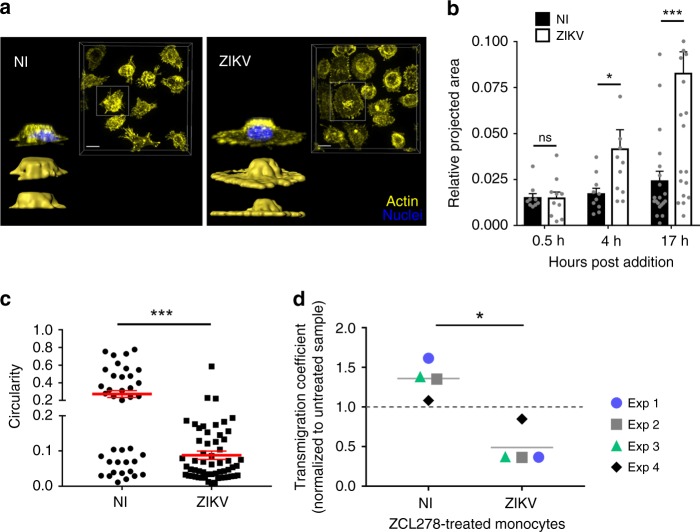
ZIKV induces a spread-out morphology of monocytes. **a** Noninfected (NI) or ZIKV-infected monocytes were plated in wells coated with fibronectin. Upon fixation, cells were permeabilized and stained with Phalloidin A568 (yellow) and Dapi (blue). Images were acquired with a spinning-disk confocal microscope. For each condition, the images show a top view of the unprocessed fluorescence signal of a field of view (upper right) and isosurface-processed 3D reconstructions in the side view (down left). Scale bar: 10 µm. **b** ZIKV-infected or noninfected (NI) monocytes from two healthy donors were added to hCMEC/D3 and processed as mentioned in Fig. 5g. The relative projected area (area covered by the surface of individual monocytes) was measured at the indicated times post monocyte addition to the endothelial layer. Each bar graph corresponds to an experiment performed on monocytes from two donors showing the mean +/− SEM from two individual experiments. **c** Circularity of individual monocytes was measured 17 h after monocyte–hCMEC/D3 coculture by using ImageJ. Each dot corresponds to a single monocyte and the red bars correspond to the mean ± SEM from two individual experiments from two donors. A circularity value of 1 indicates a perfect circle and as the value approaches 0, it indicates an increasingly elongated polygon. **d** A transmigration assay in a transwell was performed with noninfected (NI) or 48 h ZIKV-infected (ZIKV) monocytes in the presence or absence of 50 µM ZCL278. The ratio of transmigrating cells between ZCL278-treated and untreated samples is shown. Each dot corresponds to an individual donor. Two-tailed *p* value was nonsignificant (ns), <0.05 (*) or <0.0005 (***). Statistical significance was determined by using a *t* test. Source data in **b**–**d** are provided as a Source Data file

From the imaging in Fig. 5g and 3D reconstructions in Fig. 6a, we noticed that exposure to ZIKV correlated with an extended spread morphology of the monocytes. Quantification of the cell’s area and circularity upon attachment to endothelial cells in ZIKV-exposed monocytes confirmed that ZIKV induces a dramatic spread-out morphology compared with naive monocytes (Fig. 6b, c and Supplementary Fig. 9).

Given the importance of Cdc42 on integrin-mediated cell adhesion, spreading, and transmigration^38,39^, we tested the small molecule ZCL278, in which activity was initially reported to suppress Cdc42-dependent cell motility^28^, as an inhibitor of ZIKV-induced transmigration. Remarkably, ZCL278, when added post infection, selectively prevented transmigration of monocytes exposed to ZIKV (Fig. 6d), while noninfected cells were insensitive to the drug.

Together, our data show that ZIKV induces higher adhesion, spreading, and transmigration of human primary monocytes across an in vitro BBB-like model.

### ZIKV-exposed monocytes exhibit higher transmigration properties in vivo

**Fig. 7:**
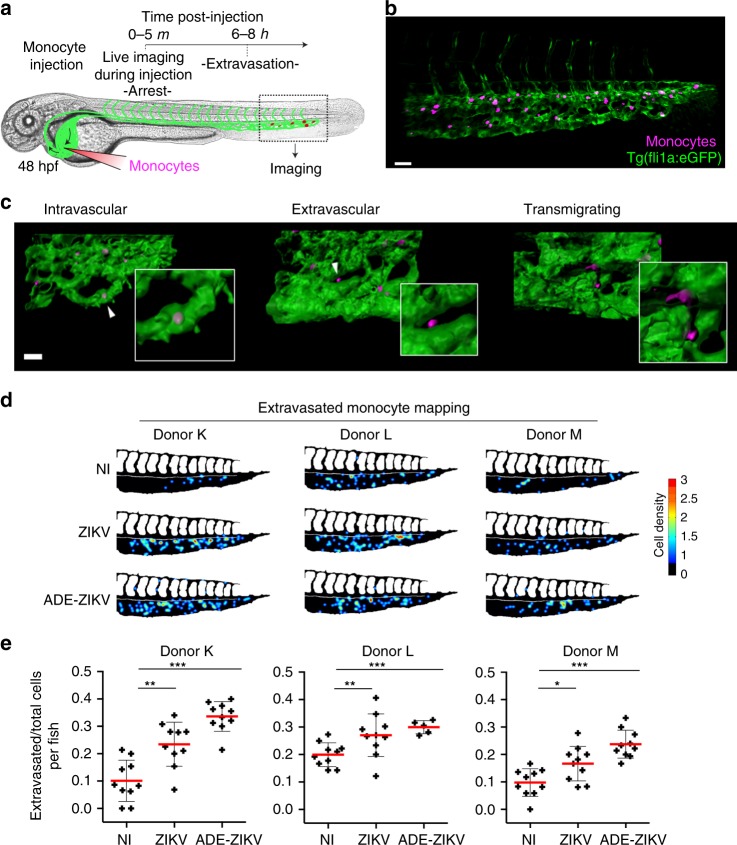
ZIKV enhances monocyte transmigration in zebrafish embryos. **a** Representative scheme of the experimental design associated with the zebrafish model. Human primary monocytes were noninfected (NI), or infected for 48 h with ZIKV in the absence (ZIKV) or presence (ADE–ZIKV) of 20 ng/mL of the 4G2-enhancing pan-*Flaviviridae* antibody (see Supplementary Fig. 10), stained with CellTrace, and injected into the duct of Cuvier of *Tg(fli1a:eGFP)* zebrafish embryos (GFP-labeled endothelial cells^44^). **b** Zebrafish imaging was done at 6–8 h post injection by scanning confocal microscopy. Representative 3D confocal image of the monocytes’ distribution within the tail of a zebrafish embryo vasculature (associated with Supplementary Movie 1) at 6 h post injection. Scale bar: 40 µm. **c** Three-dimensional reconstruction of the endothelium (green) and monocytes (magenta) that remained in the bloodstream (left panel, intravascular), transmigrated (middle panel, extravascular), or in the process of transmigrating (right panel). Scale bar: 25 µm. **d** Cell dispersion was manually counted and localized in the caudal plexus by using the stereotype patterning of intersegmental vessels (ISVs) as a reference. The data were compiled to generate heatmaps by using a custom-made MATLAB plugin. Representative heatmaps are represented for fish injected with monocytes from three individual donors. **e** Quantification of the mean ± SD of the ratio of extravasated monocytes (from the three donors used in **d** at 6–8 h post injection. Each cross represents the ratio calculated from all the monocytes tracked within a fish. Acquisition of the different conditions was performed in random order. Two-tailed *p* value < 0.05 (*), <0.005 (**), and <0.0001 (***). Statistical significance was determined by using a *t* test. Source data in  **e** are provided as a Source Data file

We next aimed at demonstrating the relevance of this phenotype in vivo. Since transmigration is a very challenging process to study in mammals, we tested whether zebrafish embryos could represent a suitable alternative. To this end, we developed a novel quantitative xenotypic system based on a procedure designed to dissect the early behavior of circulating cells in the zebrafish embryo bloodstream^40,41^. Monocytes were infected with ZIKV alone or ZIKV in the presence of a pan-flavivirus antibody known to increase infection through antibody-dependent enhancement (ADE)^42,43^ (Supplementary Fig. 10). Transgenic zebrafish embryos *Tg(fli:EGFP)* modified to express EGFP in endothelial cells^44^ were injected with prelabeled human primary monocytes (see Fig. 7a, b, Supplementary Movie 1, and Materials and Methods for details) and imaged ≈6 h post injection, a timing sufficient to observe the monocytes inside, outside, and transmigrating through the vasculature (Fig. 7c). Mapping the location of the monocytes that successfully transmigrated out of the vascular caudal plexus did not show significant differences between the different conditions (Fig. 7d). However, the percentage of extravasated cells was significantly increased when monocytes were previously exposed to ZIKV for all three donors tested (Fig. 7e). Transmigration efficiency was not linked to the percentage of ZIKV-infected cells as ADE–ZIKV had no significant further effect on monocyte transmigration compared with monocytes infected in the absence of ADE.

### ZIKV-exposed monocytes exhibit higher arresting properties in vivo

**Fig. 8:**
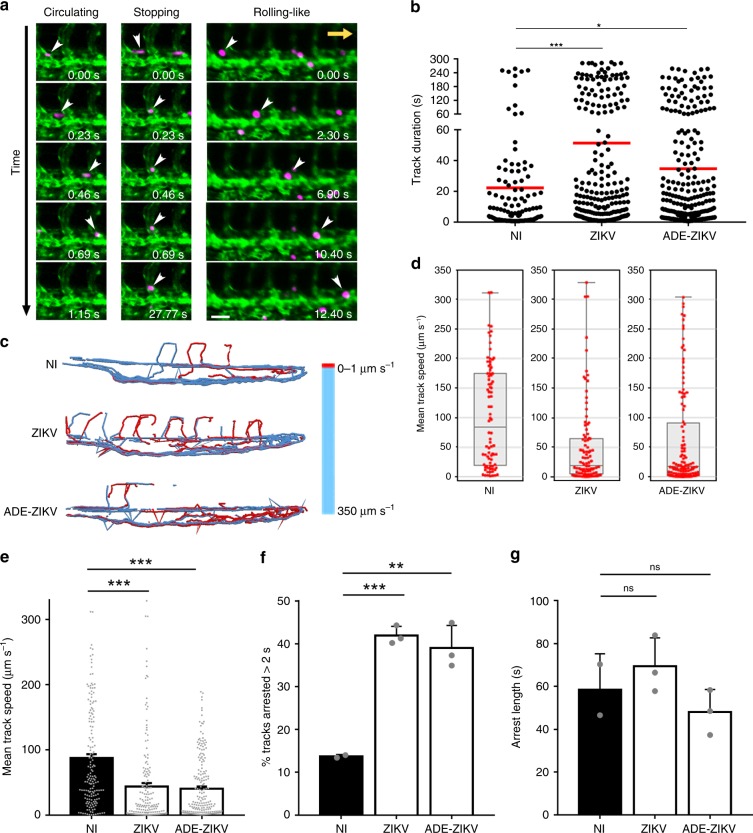
ZIKV favors monocyte arrest in zebrafish vessels, but does not affect their arresting time. Human primary monocytes from three donors were treated and injected as in Fig. 7a, and imaged with an epifluorescence microscope immediately after injection (0–5 min). **a** Micrographs representing the type of movement observed (Supplementary Movie S2). White arrowheads indicate examples of monocytes circulating (left panels), stopping (middle panels), or undergoing a rolling-like movement (right panels). Note that timescales are different for each behavior. The yellow arrow represents the direction of the bloodstream. Scale bar: 40 µm. **b**–**g** Monocytes were automatically tracked over time. **b** The track duration (in seconds) of each monocyte was quantified. Each dot corresponds to a single monocyte tracked over ≈5 min and the red line represents the mean (NI *n* = 2 fish; ZIKV *n* = 3 fish; ADE–ZIKV *n* = 3 fish). **c** Color-coded representation of monocyte speed in the zebrafish vasculature. Red tracks indicate arrested monocytes. **d**, **e** Quantification of the mean track speed of monocytes. Each red dot corresponds to a single monocyte tracked over ≈5 min. **d** The box plots are representative of the data of one fish. The center line is the median, the box extends from 25th to 75th percentiles, and the whiskers include from the smallest to the largest value. **e** The bar graph shows the mean ± SD of NI *n* = 2 fish, ZIKV *n* = 3 fish, and ADE–ZIKV *n* = 3 fish per condition. Each dot is one tracked monocyte. Quantification of **f** the percentage and **g** the arrest length (in seconds) among the monocytes that arrested for more than 2 s. **f**, **g** The bar graphs represent the mean ± SEM. Each dot represents the mean of all monocytes tracked in a given fish (NI *n* = 2 fish (139 tracks), ZIKV *n* = 3 fish (269 tracks), and ADE–ZIKV *n* = 3 fish (316 tracks)). Two-tailed *p* value was ns = nonsignificant, <0.05 (*), <0.005 (**), and <0.0001 (***). Statistical significance was determined by using a *t* test. Source data in **b**, **e**, **g** are provided as a Source Data file

To further determine the mechanism by which ZIKV promotes monocyte transmigration in vivo, we defined the hemodynamic behavior of circulating human monocytes in the bloodstream of zebrafish embryos upon injection. Three main dynamic phenotypes could be monitored: circulating, stopping, and rolling-like motion (Fig. 8a and Supplementary Movie 2). Automated tracking of circulating monocytes revealed that previous exposure to ZIKV significantly increased monocyte track duration (Fig. 8b). In parallel, the speed of ZIKV-exposed monocytes was drastically diminished (Fig. 8c–e). These measurements correlated with a greater percentage of ZIKV-exposed monocytes immobilized onto the endothelial vasculature compared with monocytes that were not in contact with the virus (Fig. 8f). However, once immobilized on the vessel wall, the average time of monocyte arrest was independent of the infection status of the cell (Fig. 8g). Of note, enhancing ZIKV infection of monocytes through ADE did not potentiate these behaviors.

These data suggest that exposure to ZIKV promotes the initial attachment of human primary monocytes to the endothelial cell wall without impacting their arrest duration.

### Transmigrated monocytes disseminate ZIKV to cerebral organoids

**Fig. 9:**
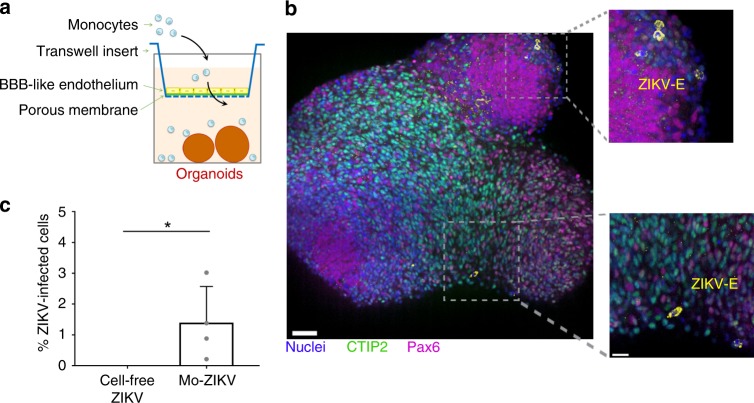
Transmigrated ZIKV-infected monocytes promote viral dissemination to cerebral organoids. **a** Representative scheme of the experimental design. Neural organoids were located under a transwell where hCMEC/D3 cells were previously grown for 7 days. On top of a transwell, ZIKV-infected monocytes from two healthy donors, not-infected monocytes, cell-free ZIKV, or mock were added. After an overnight incubation, the transwells were removed and the organoids were cultured for 2 or 9 days, and then they were fixed and processed for **b** immunofluorescence or **c** flow cytometry. The bar graph shows the mean of an experiment performed in duplicate ± SD. Two-tailed *p* value < 0.05 (*). Statistical significance was determined by using a *t* test. Scale bar: 50 µm. Source data in **c** are provided as a Source Data file

We then intended to directly link monocyte transmigration to viral dissemination and CNS neuroinvasion. We thus established a coculture transmigration assay, in which transwells coated with an endothelial monolayer of hBMEC/D3 cells, were incubated with cell-free ZIKV or ZIKV-associated monocytes in the top chamber, and with 6-wpd cerebral organoids in the bottom chamber (scheme Fig. 9a). In this setting, and in the absence of chemokine addition in the organoid-containing bottom chamber, we observed that the proportion of monocytes that transmigrated was higher when exposed to ZIKV than in the absence of virus (Supplementary Fig. 11a), consistent with our previous observations indicating that ZIKV enhances monocyte transmigration (Fig. 4). The transwell inserts were removed at the end of the overnight transmigration process, at a time that did not allow infection of endothelial cells (Fig. 4c), and the cerebral organoids were collected 48 h later. Flow cytometry analysis of the dissociated organoids revealed that the cerebral cells were more permissive to infection driven by ZIKV-infected monocytes than by cell-free virus (Fig. 9b, c). Nevertheless, at 9 dpi, no difference in the percentage of cerebral-infected cells was observed (Supplementary Fig. 11b), suggesting that cell-free virus can cross the endothelium in the absence of leukocyte carriers, but with delayed viral dissemination.

Together, these data indicate that ZIKV-induced monocyte transmigration promotes viral dissemination across the vessel wall.

## Discussion

In this study, we propose a model in which ZIKV productively infects a subset of blood-circulating monocytes and induces phenotypical and morphological changes, which in turn leads to higher monocyte adhesion and transmigration abilities. Upon transmigration, monocyte-associated ZIKV shows faster infectiveness kinetics toward reconstituted *mini-brains* than cell-free virus.

We have shown that cells derived from circulating monocytes are host targets in the brain of a ZIKV-infected human fetus. Resident perivascular macrophages (CD163^+^) and microglia (CD68^+^) originate from the embryonic yolk sac or from blood-circulating monocytes that differentiate upon transmigration^45,46^. The importance of the recruitment of circulating monocytes to the CNS during the course of an infection by West Nile Virus (WNV), a ZIKV-related Flavivirus, was previously demonstrated in mice^47^. This study indicates that the infiltrated myeloid cells in the CNS originate from blood-circulating monocytes. A recent study showed that the recombinant NS1 protein of several Flaviviruses induces transient endothelial permeability^48^. This work highlighted the presence of vascular leakage in mice injected with recombinant NS1, but further in vivo studies by using ZIKV infection models that recapitulate the physiopathology of a natural infection in humans will be required. Negligible BBB disruption was found in WNV-infected mice with encephalitis^47^ and our in vitro data failed to detect endothelial permeability following ZIKV exposure (Fig. 4b), similarly to previously published data^26^. Moreover, ZIKV is mostly asymptomatic in humans and does not induce hemorrhagic fever or cytokine storm^4,49^, suggesting that endothelial leakage may not be a major feature of ZIKV physiopathology.

Endothelial cells have also been reported to be targets of ZIKV in vitro^24–26^ by using different viral strains and/or cell lines. However, we did not observe infection or disruption of endothelial cells from the BBB of a naturally ZIKV-infected human fetus with microcephaly (Fig. 1e) and in our experimental design, hCMEC/D3 cells were not infected by ZIKV (Fig. 4c). Thus, the virus’ main access to the CNS remains to be further characterized as several models, including endothelial cell infection, the Trojan horse hypothesis, and transient BBB permeability remain plausible and not mutually exclusive. Further work will also be required to assess whether ZIKV could subvert other pathways, such as transcytosis, to cross the BBB.

We and others have observed that ZIKV does not induce a robust immune response in monocytes (our proteomics data in Fig. 5a, b and refs. ^50–52^), in sharp contrast to DENV2 and HIV-1^53,54^. It could be explained by the capacity of the NS5 protein of ZIKV to suppress interferon signaling^55^. However, ZIKV infection is not fully immunologically silent in all cell types and higher production of MCP-1, a monocyte chemoattractant, in mice and macaques has also been reported^56,57^. Moreover, the work by O’Connors et al. established that monocytes are targeted by the virus in macaques and that these cells are recruited to tissues, promoting viral persistence^56^. Therefore, ZIKV-infected monocytes could represent a bona fide Trojan Horse-like carrier used by ZIKV to cunningly disseminate into the CNS. Therefore, monocytes may represent an important systemic mediator of tissue invasion and viral persistence, and additional in vivo studies will help to evaluate the relative contribution of monocytes in the ZIKV neuroinvasion process.

ZIKV enhances monocyte adhesion and transmigration through the vessel wall, inducing higher expression of adhesion molecules at the monocyte surface (Fig. 4). This mechanism of action is specific to ZIKV as we did not observe the adhesion-related pathway upregulation in HIV-1 and DENV2 infection of monocytes. HIV-1 also enhances transmigration to the CNS^58^, but the underlying molecular mechanism likely differs from the one of ZIKV, as HIV-infected monocytes do not adhere significantly more than control cells (Fig. 5f–h). Dengue virus does not trigger transmigration of J774 monocytic cells^59^, further highlighting the specificity of ZIKV–monocyte interactions that we reported here. The impact of ZIKV-infected monocytes on the adhesion molecule profile of endothelial cells would also be relevant to address in future work, as monocyte–endothelial communication plays an important role in adhesion^60^.

Transmigration induced by ZIKV was prevented by treating cells with an inhibitor of Cdc42 function (Fig. 6d). A previous report pointed out that Cdc42 was not required for physiological monocyte adhesion and spreading^61^, and thus, ZIKV could engage a Cdc42-dependent actin remodeling program that is not usually implicated in physiological monocyte transmigration. This observation may have important implications for the design of treatments with selective activity. The dual action of ZCL278 as a Cdc42 inhibitor and as a potent antiviral molecule may be a promising antiviral option that requires further investigations.

Live-cell imaging of leukocyte circulation, arrest, rolling, and transmigration is very challenging to perform in complex mammals, such as in mice. The technical limitations include body hindrance under the microscope, heartbeat-repetitive pulses, lack of organ transparency, and loss of the injected cells into body parts that are not accessible to imaging. Although powerful 3D organoid models that mimic blood vessels are being developed^62^, they represent in vitro systems that do not fully recapitulate the complexity of a living organism. In this context, the zebrafish embryo represents a promising in vivo alternative, previously used to study mammalian viruses^63–65^. Indeed, the zebrafish embryo is relatively transparent, easy to handle, and displays fast growth kinetics, and numerous tools for genetic manipulation are available. Moreover, the zebrafish embryo represents a classical approach to study cancer cell extravasation^66,67^, but has never been employed, to our knowledge, to study virus-induced transmigration. Here, we have developed a xenotypic system, injecting human primary monocytes into zebrafish embryos (Figs. 7 and 8). We showed that human primary cells retained their capacity to undergo diapedesis through a zebrafish endothelial barrier, suggesting that active molecular interactions between these cells are taking place despite the species’ gap. This experimental in vivo model was recently exploited to perform extensive human monocyte phenotyping upon injection in zebrafish^68^. The development of this robust and easy-to-manipulate in vivo model allowed quantitative analyses of monocyte transmigration under hemodynamically relevant conditions that would not have been achieved with other animal models.

The cerebral organoid model derived from hESCs was previously characterized and used in the context of ZIKV infection^69^ as these organoids recapitulate much of the architecture of the fetal brain, modeling whole-brain development^70^. A limitation however is that cerebral organoids do not spontaneously develop a functional vasculature (unless transplanted^71^), and we thus had to engineer a transwell-organoid system to allow the combined investigations of both transmigration and dissemination (Fig. 9). In this system, we found that cell-free virus had slower kinetics of dissemination than monocyte-associated virus, suggesting that monocytes could accelerate viral spreading toward tissues.

In conclusion, by using various in vitro, ex vivo, and in vivo models, we have shown that ZIKV enhances monocyte transmigration to efficiently cross endothelial barriers and promotes viral dissemination. Our findings are of importance in regard to our current understanding of ZIKV–host interactions and highlight monocyte transmigration as a process that could be specifically targeted for therapeutic intervention.

## Methods

### Cell culture

Purified human primary monocytes were cultured in RPMI 1640 (Gibco) supplemented with 2% FBS (Dutscher) and 1% Penicillin–Streptomycin (Gibco). Cells were cultured into round-bottom tissue culture-treated 96-well plates. The human Cerebellar Microvascular Endothelial Cells D3 (hCMEC/D3) cells^34^ (provided by S. Bourdoulous, Institut Cochin, France) were cultured by using EndoGRO‐MV Complete Culture Media (Millipore) supplemented with 1 ng/mL bFGF (Sigma) and 1% Penicillin–Streptomycin (Gibco). Cells were plated and cultured in dishes coated with rat collagen-I (R&D Systems). Lithium Chloride (10 mM) and Resveratrol (10 μM) were added when seeding the cells in the culture inserts (cf. transmigration assay). Vero cells (ATCC, CCL-81) were cultivated in High-glucose DMEM media with L-Glutamine (Hyclone), 10% FBS, and 1% Penicillin–Streptomycin. All cell types were maintained at 37 °C in a humidified atmosphere containing 5% CO_2_.

### Human primary monocytes

The buffy coats were obtained from healthy blood donors (EFS Strasbourg, France). All donors signed informed consent allowing the use of their blood for research purposes. PBMCs were purified on a Ficoll density gradient (GE Healthcare). Monocytes were isolated by positive selection by using CD14^+^ microbeads and magnetic columns from Miltenyi Biotec. Monocytes were derived from a total of 22 individual blood donors. For experiments shown in Figs. 3b, 4b, c, 5f, h, 6d, and 9c, donors were randomly chosen and cannot be compared between experiments. The source data underlying these figures are referred to “datasets” in the Source Data file. For experiments shown in Figs. 2a, b, d, and 7e, the same donors were used and are labeled by letters (A, B, C, …) for direct comparison between experiments.

### Viruses

The following ZIKV strains were obtained through BEI Resources, NIAID, NIH: Zika Virus, FLR, NR-50183 (ZIKV^C^) and ZIKV, H/PAN/2015/CDC-259359, NR-50219 (ZIKV^P^). ZIKV^C^ and ZIKV^P^ were isolated in December 2015 from the blood of infected patients from Colombia and Panama, respectively. The ZIKV strain PRVABC59, which was used as a control for immunohistochemistry experiments, originated from a human serum specimen in December of 2015 in Puerto Rico (NCBI Accession No. KU501215). ZIKV^C^ and ZIKV^P^ were grown on Vero cells for 72 h. The cleared supernatant was stored at –80 °C and used for infection experiments. To obtain concentrated virus, the ZIKV-containing supernatant was passed through a 0.45-µm filter (Millipore) and ultracentrifugation was performed at 30,000 *g* for 15 h by using a SW28 rotor (Beckman Coulter). In all experiments, supernatants from noninfected Vero cells were used as noninfected controls (mock). The multiplicity of infection (MOI) was calculated by virus titration on Vero cells by using standard plaque assay to measure plaque-forming units per mL of virus (PFUs/mL). All experiments involving infectious ZIKV were performed in a BSL-3 facility. DENV2 strain 16681 was obtained from the Biological Resource Center of Institut Pasteur, France. Viral stocks were prepared on C6/36 cells (provided by M. Flamand, Institut Pasteur, France). Virus titers were assessed by focus-forming assays on Vero cells^27^. HIV-1 was obtained from the supernatant of HEK 293T cells transfected with pNL(AD8)^72^.

### Flow cytometry

To process the cells for flow cytometry analysis, the samples were first blocked with 10% FBS in PBS. Human FcR Blocking Reagent (Miltenyi Biotec) was added to prevent unspecific binding of IgGs to Fc receptors (FcR). To label cell surface markers, samples were stained with a cocktail of primary-labeled antibodies at 4 °C. To detect intracellular proteins, cells were fixed in 4% paraformaldehyde (PFA) and permeabilized by using 0.1% Triton X-100 (Sigma) and 0.5% BSA (Euromedex). PBMC samples were acquired with a 10-channel Navios flow cytometer (Beckman Coulter) with a combination of 405-, 488-, and 640-nm lasers. The other samples were acquired with a LSR II flow cytometer (BD Biosciences), a MACS Quant flow cytometer (Miltenyi Biotec), a Cytoflex flow cytometer (Beckman Coulter), or a Novocyte (ACEA Biosciences). All data were analyzed by using FlowJo, LLC software version v10.4.2.

### Antibodies

A list of the used antibodies with their dilutions can be found in Supplementary Table 1. When necessary, some primary antibodies were labeled with Alexa Fluor 488 or 647 by using an antibody labeling kit (Molecular Probes).

### Reagents

ZCL278 was purchased from Tocris and diluted in DMSO at 50 mM. ICAM-1 recombinant protein (#ADP4-050), and fibronectin isolated from human plasma (1918-FN-02M) were purchased from R&D Systems. The type I solution of rat collagen, Lucifer Yellow CH dilithium salt, Resveratrol, and Lithium Chloride were purchased from Sigma-Merck. The recombinant human CCL2/MCP-1 Protein was obtained from R&D Systems and Miltenyi Biotec.

### Human subjects

The human fetal brain tissues used in this study were obtained from a PCR-confirmed case of congenital ZIKV with a gestational age of 22 weeks, similar to cases that we previously described^73^. The fetal brain tissues were initially submitted to CDC for diagnostic testing during the Zika response as part of the routine pathology services provided by CDC to state and territorial authorities. Both the initial receipt of human samples [061116SZ] and the secondary use [102517GH] were submitted for review human subjects in accordance with standard NCEZID procedures, and both were determined to be outside the scope of IRB review requirements under 45 CFR 46 [pre-2018 rule] by the NCEZID Senior Human Subjects Advisor, as authorized by CDC institutional policy, as the fetuses are not living individuals [46 CFR 46.102(d), pre-2018 rule].

### Immunohistochemistry

Immunohistochemical assays were performed by using a peroxidase and alkaline-phosphatase detection system. Briefly, 4-μm tissue sections were placed on slides. The sections were deparaffinized in xylene and rehydrated through graded alcohol solutions. Colorimetric detection of antibodies was performed by using the EnVision G|2 Doublestain System Rabbit/Mouse (DAB + /Permanent Red) (Agilent). Heat-induced epitope retrieval (HIER) by using EDTA-based buffer was performed (Biocare Medical) using the Biocare NxGen decloaker program #5 (110 °C for 15 min). All slides were then treated according to the Dako EnVision G|2 Doublestain System procedure. Slides were incubated in Endogenous Enzyme block for 5 min, primary antibody for 10 min, HRP-Polymer for 10 min, and DAB working solution for 10 min. Doublestain block was then applied for 3 min, and the second stain procedure consisted of the other primary antibody for 10 min, followed by the Rabbit/Mouse Link, AP-Polymer, and Permanent Red working solution for 10 min each. Slides were double stained with a ZIKV-specific anti-NS1 monoclonal antibody (clone 3C2) and one of the following markers: CD68, CD163, CD14, or CD45. In each double stain, CD markers and ZIKV were each labeled with Permanent Red and DAB in serial sections. Appropriate negative control serum was run in parallel. Slides were counterstained in Mayer’s Hematoxylin (Polyscientific) and blued in Lithium carbonate (Polysciences). Slides were coverslipped with aqueous mounting medium (Polysciences). All steps of the staining procedure, with the exception of the HIER, were performed at room temperature.

### Human cortical organoids

Cortical organoids were generated from H9 human embryonic stem cells (hESCs; female, WA09, WiCell)^31^. Briefly, H9 hESCs were grown on mitomycin-C-treated MEF feeding layers in ESC culture medium: DMEM/F12 + l-glutamine (Thermofisher) with 20% KnockOut serum replacement (Thermofisher), 1× non-essential amino acids (Thermofisher), 100 μM β-mercaptoethanol (Thermofisher), and 1× Penicilin–Streptomycin (Thermofisher). The hESC culture medium was changed daily and freshly supplemented with 8 ng/ml human bFGF (Sigma). For organoid differentiation, the culture medium was replaced with differentiation medium: ESC culture medium with 1× Sodium Pyruvate (Thermofisher) but without addition of human bFGF. Colonies of ~2 mm in diameter were manually lifted by using a Corning cell lifter and were transferred to a 60-mm ultra-low attachment dish (Corning). After 24 h (day 0), embryoid bodies had formed and half of the medium was replaced with differentiation medium supplemented with 3 μM IWR-1-Endo (Sigma), 1 μM Dorsomorphin (Sigma), 10 μM SB-431542 (Sigma), and 1 μM Cyclopamine (Sigma). The medium was replaced every other day until the harvest. From day 3 onward, 60-mm dishes with organoids were placed on a belly-button shaker in the incubator. From day 18 onward, organoids were cultured in Neurobasal medium (Thermofisher) with 1× N2 supplement (Thermofisher), 2 mM l-glutamine, 1× pen–strep, and 1 μM Cyclopamine. From day 24 onward, Cyclopamine was not added anymore.

Cerebral organoids were cocultured with ZIKV-infected monocytes, noninfected monocytes, cell-free ZIKV, or mock for either 2 or 9 days. The media was replaced every other day. At the indicated time points, the organoids were processed for flow cytometry or immunofluorescence. For the flow cytometry analysis, the organoids were dissociated with trypsin, stained with Viobility 405/502 (Miltenyi), fixed with 4% PFA for 20 min at RT, and permeabilized and stained with anti-CTIP2, Pax6, CCR2, and 4G2 antibodies. For immunofluorescence, the monocytes were prelabeled with CellTrace Violet (Invitrogen) before addition to the organoids. The cerebral organoids were then fixed with 4% PFA for 1 h at RT, permeabilized with 1% Triton X-100 (2 days at RT), and labeled with anti-CTIP2, Pax6, and 4G2 antibodies (1 day at RT), and then with fluorescent secondary antibodies and Dapi (1 day at RT). They were clarified overnight at RT by using RapiClear 1.52 reagent (Sunjin Lab). The clarification was observed to be important to perform in-depth whole-organoid imaging, as the clarification medium homogenizes the refractive index of the organoid and diminishes scattering of the emitted photons. All stainings were done in the presence of human FcR-blocking reagent. Image acquisition was performed on a spinning-disk confocal microscope (Dragonfly, Oxford Instruments) equipped with an ultrasensitive 1024 × 1024 EMCCD camera (iXon Life 888, Andor) and four laser lines (405, 488, 561, and 637 nm). A 20×, NA 0.8 air objective was used for whole-organoid imaging and a 40×, NA 1.15 (Nikon) water-immersion long-distance (0.6 mm) objective was used for in-depth imaging of selected areas of the organoid. For the z-stacks a z step of 1 μm was used. Images were processed by using Imaris ×64 (Bitplane) version 9.2.

### Organotypic cultures of mouse cerebellar slices

Organotypic cerebellar slices were prepared from P10–P12 CD1 mice^74^. This study was carried out in strict accordance with the national and European community guidelines for laboratory animal welfare and experimentation. All experiments were approved in advance by the Ethics Committee of Strasbourg, France (CREMEAS, CEEA35). Mice were bred and housed in a 12-h light/dark cycle with free access to food and water. After decapitation, the cerebellum was dissected out in minimum essential medium (MEM) with 25 mM HEPES, 4 g/L D-Glucose, 50 UI/mL penicillin, and 50 μg/mL streptomycin. Isolated cerebella were cut into 350 -µm parasagittal slices by using a McIlwain tissue chopper (MickleLab). Slices were transferred onto 30-mm culture inserts with 0.4-µm pore size (Millicell, Merck Millipore). Inserts were placed in six-well plates containing 1 mL of culture medium per well. Culture medium was composed of 50% MEM with 25 mM HEPES, 25% Basal Medium Eagle, and 25% heat-inactivated horse serum supplemented with 2.10−3 M GlutaMAX, 50 UI/mL penicillin, 50 μg/mL streptomycin, and 4 g/L D-glucose. The slices were incubated at 37 °C in a humidified incubator equilibrated with 95% air/5% CO_2_. The medium was refreshed every 2 days. d-Glucose, penicillin, and streptomycin were from Sigma-Aldrich. Culture media, horse serum, and GlutaMAX were from Thermo Fisher.

One week after culture, the slices were treated with 10^6^ monocytes (ZIKV-infected or not) per slice. The cell-free virus condition was performed by using 10^6^ PFUs of ZIKV, corresponding to the amount used to infect monocytes at a MOI of 1. Monocytes and virus preparations were applied dropwise to the top (air interface) of each slice.

Individual organotypic cerebellar slices were fixed by using 4% PFA in phosphate buffer solution (PBS) for 30 min at room temperature. Then, the slices were transferred to a saturation solution containing PBS with 0.2% Triton, 2% bovine serum albumin (Sigma-Aldrich), and 10% goat serum (Merk Millipore) for a minimum of 12 h. Purkinje cells were specifically labeled by using an anti-calbindin antibody. The secondary antibody was an Alexa-488 donkey anti-mouse. Primary and secondary antibodies dilutions were prepared in PBS supplemented with 0.2% Triton, 2% bovine serum albumin (BSA), and 5% normal goat serum (Merck Millipore), and labeling at each step was performed for a minimum of 24 h at 4 °C. Slices were mounted on glass coverslips with Prolong Gold antifade reagent (Invitrogen). Confocal images were produced at the imaging facility service of the Neuropole (CNRS, UPS 3156, Strasbourg, France) by using a Leica SP5II inverted confocal microscope (10×, 20×, or 63× objectives). Fields were acquired by using a 488-nm argon laser and a 561-nm diode-pumped solid-state laser.

### ZIKV RNA detection by FISH

Cells were attached on #1.5 glass coverslips by using Poly-l-lysin, and FISH was performed by using the QuantiGene ViewRNA ISH Assay Kit following the manufacturer’s instruction (Affymetrix) and a specific probe recognizing ZIKV plus strand RNA (Affymetrix VF1-19981). Cell DNA was stained with NucBlue (Thermo Fisher). Images were acquired with an inverted confocal microscope (Zeiss LSM 700) and processed with Zen2 (blue edition) software (Zeiss).

### RT-qPCR

Total RNA was extracted from monocytes with the NucleoSpin RNA II kit (Macherey-Nagel). First-strand complementary DNA synthesis was performed with the SuperScript III First-Strand Synthesis System Reverse Transcriptase (Invitrogen). Quantitative real-time PCR was performed on a real-time PCR system (ABI PRISM 7900HT) with SYBR Green PCR Master Mix (Life Technologies). Data were analyzed with the 2-DDCT method^75^, with all samples normalized to GAPDH. The ZIKV forward and reverse primers used were 5′-AARTACACATACCARAACAAAGTGGT-3′ and 5′-TCCRCTCCCYCTYTGGTCTTG-3′^76^. The GAPDH forward and reverse primers used were 5′-GGTCGGAGTCAACGGATTTG-3′ and 5′- ACTCCACGACGTACTCAGCG-3′. The CD99 forward and reverse primers used were 5′-CTCTTCCCCTTCTTTCCTGTG-3′ and 5′-CAAATCCAAACCCCAACCAC-3′. The ITGAL forward and reverse primers used were 5′- TCATACACCACGTCAACCTTC-3′ and 5′-CTCTTCCATGTTCAGCCTCTG-3′.

### Transmigration assay

The hCMEC/D3 cells were seeded at a density of 40–50,000 cells/cm² in hanging cell culture inserts (PET, 5-µm pores, Millipore) pre-coated with rat collagen type 1 (Sigma). The cells were kept in culture for 7 days until ready for the transmigration and permeability assays. Infected monocytes were extensively washed in RPMI 2% FBS media before addition to the transwell inserts containing the hCMEC/D3 cells. In parallel, the media in the bottom chamber was replaced with fresh media containing 200 ng/mL of MCP-1 (Miltenyi Biotech). Transmigration was allowed to occur overnight (about 17 h). Monocytes were recovered from the top and bottom chambers of the insert and prepared for flow cytometry analysis.

### Lucifer yellow permeability assay

On day 7 of culture of hCMEC/D3 cells in the transwell, permeability was assessed by adding 50 µM Lucifer yellow in the top chamber. After 120 min at 37 °C, media was collected from the top and bottom chambers of the insert. In parallel, a calibration curve of Lucifer yellow was prepared (50, 25, 12.5, and 6.25 µM). Quantification of fluorescence was done by using a microplate reader (Mithras LB 940 Multimode Microplate Reader, Berthold Technologies) with excitation at 488 nm and emission filter at 520 nm. The permeability coefficient (Pc) was calculated as described in ref. ^35^.

### Cell viability

Cell viability was assessed by using the Pierce LDH Cytotoxicity Assay Kit (Thermofisher Scientific) according to the manufacturer’s instructions. Absorbance was measured with a Mithras LB 940 Multimode Microplate Reader (Berthold Technologies).

### Immunofluorescence

ZIKV-infected monocytes cocultured with either hCMEC/D3 cells or hNPCs grown on coverslips were fixed with 4% PFA, permeabilized with 0.1% Triton X-100 and 0.5% BSA, and labeled with primary antibodies and fluorescent secondary antibodies as necessary. The staining was done in the presence of human FcR-blocking reagent and nuclei were labeled with Dapi. Coverslips were mounted on slides by using Fluoromount Aqueous Mounting (Sigma). Image acquisition was performed on an AxioObserver.Z1 inverted microscope (Zeiss) mounted with a spinning-disc head (Yokogawa), a back-illuminated EMCCD camera (Evolve, Photometrics), and a ×63 or ×100, 1.45 NA oil objective (Zeiss). Images were processed by using FiJi (ImageJ software version 1.51 h) and the 3D reconstruction was performed by using Imaris ×64 version 9.2.

### Adhesion assay

Adhesion assays were performed either in plastic 96-well plates (flat bottom) or on glass coverslips. The coating of the substrate was done with 150 μg/mL of collagen, type I solution from rat tail (Sigma), 12.5 μg/mL of recombinant Human ICAM-1/CD54 (R&D Systems), or 5 µg/cm^2^ of human fibronectin from R&D Systems. Primary monocytes were added to the coated substrate and incubated for 2 h at 37 °C. Afterward, the cells were processed for immunofluorescence staining with Phalloidin A568 (Invitrogen), or analyzed for cell viability with the CellTiter-Glo Luminescent Cell Viability Assay (Promega).

### Scanning electron microscopy

ZIKV-infected monocytes cocultured with hCMEC/D3 cells on coverslips were washed twice with Cacodylate buffer (pre-warmed), and fixed with 2.5% glutaraldehyde for 1 h at RT. The samples were dehydrated in a graded alcohol series, coated with platinum, and imaged with a Phenom ProX Desktop scanning electron microscope (Thermo Fisher).

### Quantitative proteomics

The protein concentration of all samples was determined by using the RC-DC protein assay (Bio-Rad) according to the manufacturer’s instructions and a standard curve was established by using BSA. Five micrograms of protein lysate for each sample and a sample pool for quality control (QC) were heated at 95 °C for 5 min and concentrated on a stacking gel band. The gel bands were cut, washed with ammonium hydrogen carbonate and acetonitrile, reduced, and alkylated before trypsin digestion (Promega). The generated peptides were extracted with 60% acetonitrile in 0.1% formic acid followed by a second extraction with 100% acetonitrile. Acetonitrile was evaporated under vacuum and the peptides were resuspended in 10 µL of H_2_O and 0.1% formic acid before nanoLC–MS/MS analysis.

NanoLC–MS/MS analyses were performed on a nanoACQUITY Ultra-Performance LC system (Waters, Milford, MA) coupled to a Q-Exactive Plus Orbitrap mass spectrometer (ThermoFisher Scientific) equipped with a nanoelectrospray ion source. The solvent system consisted of 0.1% formic acid in water (solvent A) and 0.1% formic acid in acetonitrile (solvent B). Samples were loaded into a Symmetry C18 precolumn (0.18 × 20 mm, 5-μm particle size; Waters) over 3 min in 1% solvent B at a flow rate of 5 μL/min followed by reverse-phase separation (ACQUITY UPLC BEH130 C18, 200 mm × 75 μm id, 1.7-μm particle size, Waters) by using a linear gradient ranging from 1 to 35% of solvent B at a flow rate of 450 nL/min. The mass spectrometer was operated in data-dependent acquisition mode by automatically switching between full MS and consecutive MS/MS acquisitions. Survey full-scan MS spectra (mass range 300–1800) were acquired in the Orbitrap at a resolution of 70 K at 200 m/z with an automatic gain control (AGC) fixed at 3.10^6^ and a maximal injection time set to 50 ms. The ten most intense peptide ions in each survey scan with a charge state $\ge$ 2 were selected for fragmentation. MS/MS spectra were acquired at a resolution of 17.5 K at 200 m/z, with a fixed first mass at 100 m/z, AGC was set to 1.10^5^, and the maximal injection time was set to 100 ms. Peptides were fragmented by higher-energy collisional dissociation with a normalized collision energy set to 27. Peaks selected for fragmentation were automatically included in a dynamic exclusion list for 60 s. All samples were injected by using a randomized and blocked injection sequence (one biological replicate of each group plus pool in each block). To minimize carryover, a solvent blank injection was performed after each sample. A sample pool comprising equal amounts of all protein extracts was constituted and regularly injected four times during the course of the experiment, as an additional QC. Protein identification rates and coefficient of variation (CV) monitoring of this QC sample revealed very good stability of the system: 2597 of the 2805 identified proteins, namely 92%, showed a CV value lower than 25% considering all four injections.

Raw MS data processing was performed by using MaxQuant software^77^ v1.6.0.16. Peak lists were searched against a database including *Homo sapiens* protein sequences extracted from SwissProt (06-03-2019; 20 410 sequences, Taxonomy ID 9606) using the MSDA software suite^78^.

MaxQuant parameters were set as follows: MS tolerance set to 20 ppm for the first search and 5 ppm for the main search, MS/MS tolerance set to 40 ppm, maximum number of missed cleavages set to 1, Carbamidomethyl (C) set as fixed modification, and Oxidation (M) set as variable modification. False-discovery rates (FDRs) were estimated based on the number of hits after searching a reverse database and were set to 1% for both peptide spectrum matches (with a minimum length of seven amino acids) and proteins. Data normalization and protein quantification was performed by using the LFQ (label-free quantification) option implemented in MaxQuant by using a “minimal ratio count” of 1. The *Match between runs* option was enabled by using a 2-min time window after retention-time alignment. All other MaxQuant parameters were set as default. To be considered, proteins must be identified in all four replicates of at least one condition and a minimum of two unique peptides. The imputation of the missing values and differential data analysis was performed by using the open-source ProStaR software^79^. Two runs of imputation were applied, the *SLSA* mode was applied for the partially observed values and the *det quantile* for the missing in the entire condition^80^. A Limma-moderated *t*-test was applied on the dataset to perform differential analysis. The adaptive Benjamini–Hochberg procedure was applied to adjust the *p* values and FDR values under 1 or 5% depending on the comparisons were achieved. Data analyses were performed by using the STRING online tool^36^, by using the highest confidence (0.9 interaction score). The unlinked proteins were excluded from the network shown in Fig. 5a. The same data were also processed by using the GSEA online tool^37^, using features from gene ontology “biological process” and “cellular component”.

### Antibody-dependent enhancement assay

For virus–antibody complex formation, virus particles (MOI 5) were incubated for 30 min at 37 °C in the presence of serial tenfold dilutions of the 4G2 antibody in RPMI medium containing 2% FBS. The virus–antibody complexes were then added to primary monocytes and incubated at 37 °C with 5% CO_2_. At 48 hpi, the cells were thoroughly washed, fixed, and processed for flow cytometry analysis as described below.

### Zebrafish experiments

All animal procedures were performed in accordance with French and European Union animal welfare guidelines and all experiments were approved in advance by the Ethics Committee of Strasbourg (CREMEAS, CEEA35). Tg(*fli1a:eGFP*) zebrafish (*Danio rerio*) embryos (ZFIN ID: ZDB-FISH-150901-3654) were used in all experiments. Embryos were maintained in Danieau 0.3X medium (17.4 mM NaCl, 0.2 mM KCl, 0.1 mM MgSO_4_, and 0.2 mM Ca(NO_3_)_2_) buffered with 0.15 mM HEPES (pH = 7.6) and supplemented with 200 mM of 1-Phenyl-2-thiourea (Sigma-Aldrich). For all zebrafish experiments, the offspring of one single cross was selected, based on anatomical/developmental good health. Embryos were split randomly between experimental groups. Forty-eight hours post fertilization (hpf), embryos were mounted in a 0.8% low-melting-point agarose pad containing 650 mM of tricain (ethyl-3-aminobenzoate-methanesulfonate) to immobilize them. Pre-labeled primary human CD14^+^ monocytes were injected with a Nanoject microinjector 2 (Drummond) and microforged glass capillaries (20-µm inner diameter) filled with mineral oil (Sigma), and 14 nL of a cell suspension at 10^8^ cells per mL were injected in the duct of Cuvier of the embryos under the M205 FA stereomicroscope (Leica)^41^. At 5–7 h post injection, confocal imaging was performed with an inverted TCS SP5 confocal microscope with a HC PL IRAPO 20× NA 0.75 objective (Leica). The experiment was carried out three independent times by using three different monocyte donors with *n* = 10 embryos per condition (exception: *n* = 5 for the second donor, condition ADE–ZIKV). The monocytes were labeled with CellTrace Yellow (Invitrogen) according to the manufacturer’s instructions.

For live recording of the injection, a stereomicroscope with a DFC3000 G camera (Leica) was used. To capture flowing and arresting monocytes in real time, only the red channel (corresponding to injected prelabeled monocytes) was recorded during injection. After 5 min, a single image of the zebrafish vasculature was acquired and used as an overlay background for the registered time-lapse recording of monocytes.

Cell dispersion was manually counted and localized in the caudal plexus by using the stereotype patterning of intersegmental vessels (ISVs) as a reference. The data were compiled to generate heatmaps by using a custom-made MATLAB plugin^41^. Post processing was performed by using ImageJ and Imaris ×64 version 9.2 for segmentation, 3D reconstruction, and tracking.

### Reporting summary

Further information on research design is available in the Nature Research Reporting Summary linked to this article.

## Supplementary information

Supplementary Information

Description of Additional Supplementary Files

Supplementary Movie 1

Supplementary Movie 2

Supplementary Data 1

Reporting Summary

Source Data

## Data Availability

The source data underlying Figs. 2–9 (2a, b, d, 3b, e, 4b, c, e, 5b, c–e, f, h, 6b–d, 7e, 8b, 8e, 8g, 9c) are provided as a Source Data file. The complete dataset resulting from the mass spectrometry proteomics analysis has been deposited to the ProteomeXchange Consortium via the PRIDE partner repository^81^ with the dataset identifier PXD014002. All other data are available from the corresponding author upon reasonable request. A reporting summary for this article is available as a Supplementary Information file.
