# Supplementary material for: Zika virus enhances monocyte adhesion and transmigration favoring viral dissemination to neural cells
Source: Nat Commun. 2019 Sep 27;10:4430. doi: 10.1038/s41467-019-12408-x (PMC6764950; doi:10.1038/s41467-019-12408-x)
Supplement: Supplementary file 1 — Supplementary Information [file 41467_2019_12408_MOESM1_ESM.pdf]

Supplementary Information

"Zika virus enhances monocyte adhesion and transmigration, favoring viral dissemination to the CNS"

Ayala-Nunez, *et al.*

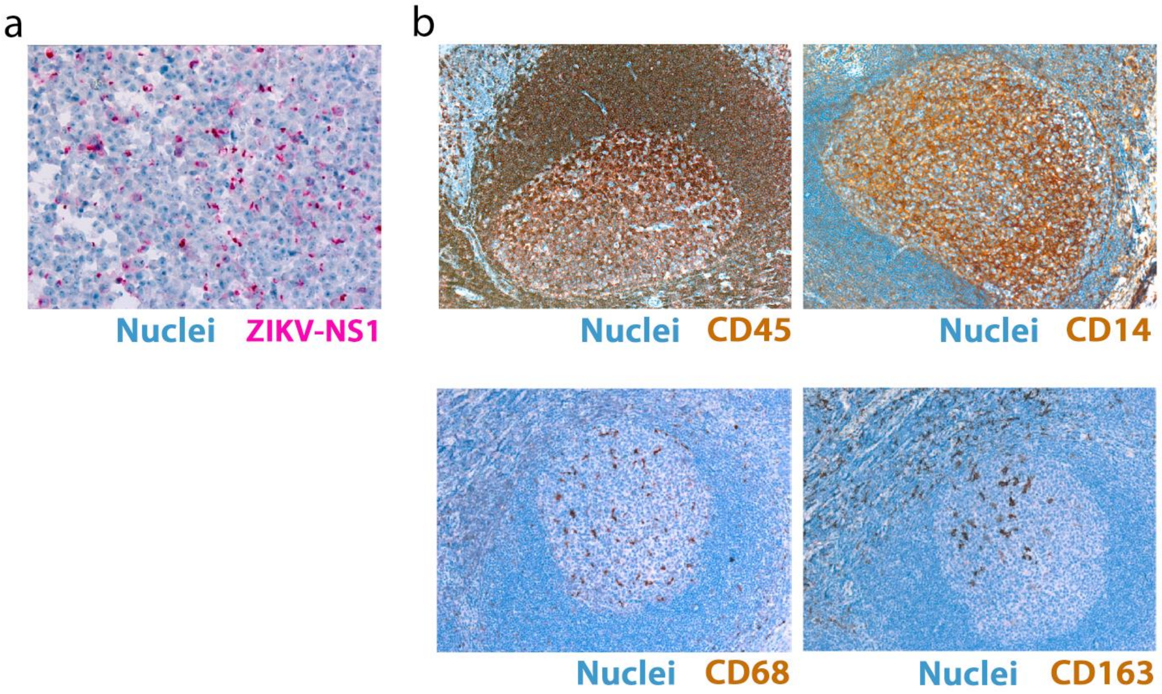

Supplemental Figure 1. Immunohistochemistry staining controls. (**a**) ZIKV strain PRVABC59 was inoculated in Vero/E6 cells. The cells were fixed and stained with anti-NS1 ZIKV 3C2 monoclonal antibody (CDC Immuno-Diagnostic Development Team). (**b**) Slices from surgical specimens of human tonsils (not infected with ZIKV) were stained for CD45, CD14, CD68 or CD163. All slides were counterstained in Mayer's Hematoxylin and blued in Lithium carbonate. Magnification: 10x.

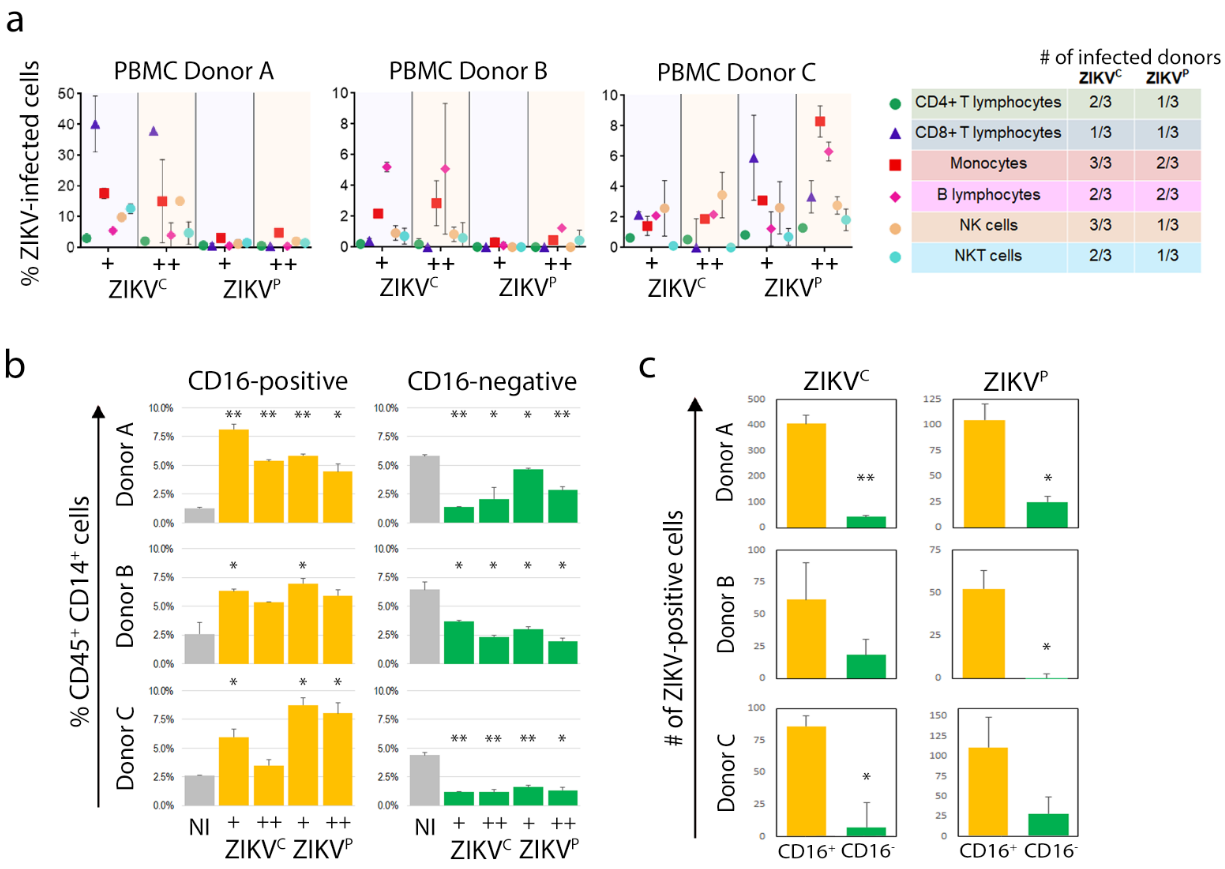

Supplemental Figure 2. Permissiveness of PBMCs to ZIKV infection. (**a**) Total PBMCs purified from 3 healthy blood donors were inoculated with $ZIKV^{C}$ or $ZIKV^{P}$ at MOI 1 (+) or 5 (++) for 48 h. Cells were stained to detect CD45, CD3, CD4, CD8, CD14, CD16, CD19, and CD56, then fixed, permeabilized, and labeled with an antibody against the non-structural viral protein NS2B. Flow cytometry analysis was performed and the $CD45^{+}$ subpopulations of $CD4^{+}$ T lymphocytes ($CD3^{+}$ $CD4^{+}$), $CD8^{+}$ T lymphocytes ($CD3^{+}$ $CD8^{+}$), monocytes ($CD14^{+}$), B lymphocytes ($CD19^{+}$), NK cells ($CD56^{+}$ $CD3^{-}$) and NKT cells ($CD56^{+}CD3^{+}$) were discriminated, and the percentage of ZIKV-infected cells ($NS2B^{+}$) were plotted as a function of the strain and MOI of ZIKV used. Each dot corresponds to the mean +/- SD of ZIKV-infected cells for each subpopulation from two individual samples. The table on the right summarizes the number of donors that showed infection according to the cell type and ZIKV strain tested. (**b**) The monocytic population ($CD45^{+}$ $CD14^{+}$) from samples described in (**a**) was further analyzed and segregated into two subpopulations of $CD16^{+}$ and $CD16^{-}$ monocytes. The percentage of $CD16^{+}$ monocytes (left panels) and $CD16^{-}$ monocytes (right panels) was quantified in cells that were not infected (NI) or infected as indicated for each donor. Compared to NI, the two-tailed *p*-value was < 0.05 (*) and < 0.005 (**) as indicated. (**c**) Among each subpopulation described in (**b**), the absolute number of infected cells ($NS2B^{+}$) was quantified for $ZIKV^{C}$ or $ZIKV^{P}$ infected monocytes at the lowest virus concentration. Comparing the number of infected cells among $CD16^{+}$ and $CD16^{-}$ monocytes, the two-tailed *p*-value is below 0.05 (*), and 0.005 (**) as indicated. Statistical significance was determined using a t-test.

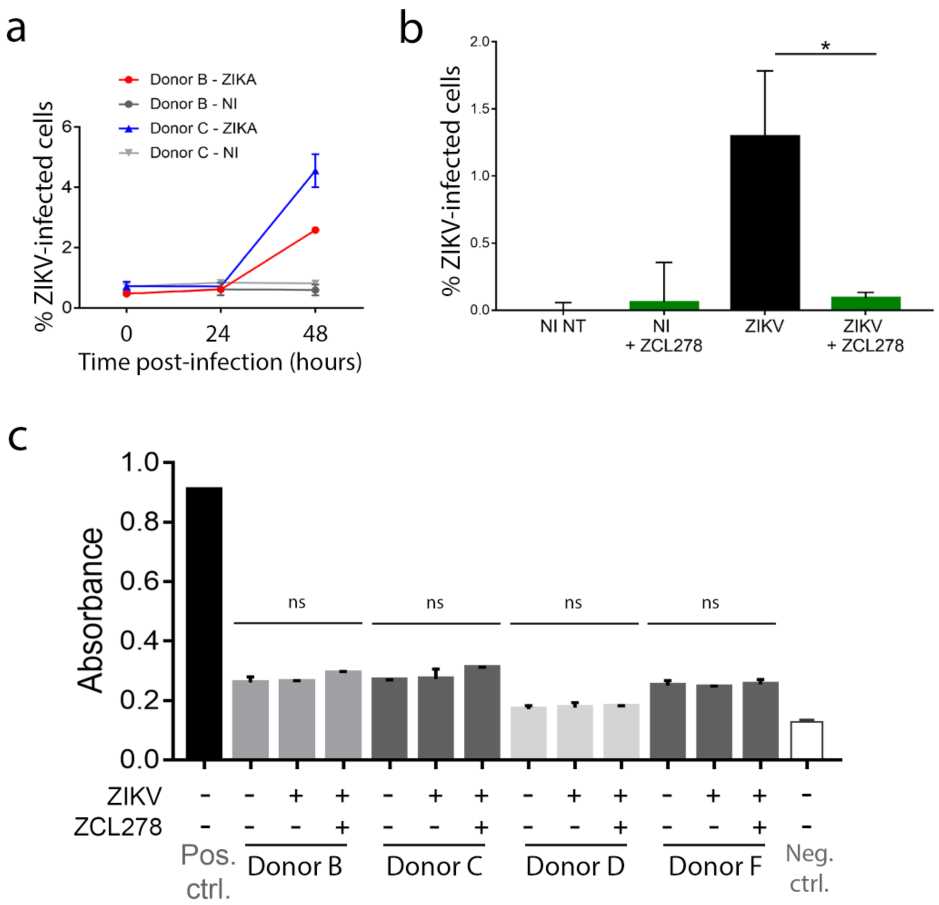

Supplemental Figure 3. Characterization of ZIKV-infected monocytes. (**a**) Monocytes from two donors were infected at MOI 1 and the percentage of ZIKV-infected cells ($NS2B^{+}$) was measured by flow cytometry at 24- and 48-hours post-infection. (**b**) Primary monocytes from two healthy donors were pre-treated with DMSO or 50 µM ZCL278 for 30 min at 37°C. The compound was then removed and the cells were infected with $ZIKV^{C}$ (MOI 1) in the presence or absence of 50 µM ZCL278. Cells were washed 4 h post-infection and incubated in RPMI 2% FBS. At 48 hpi, cells were fixed and stained and the percentage of ZIKV-infected cells ($NS2B^{+}$) was quantified by flow cytometry. The bar graph corresponds to the mean +/- SD of ZIKV-infected cells for each condition in two individual samples. The two-tailed *p*-value is < 0.05 (*). NT: non-treated, NI: non-infected. (**c**) The cytotoxicity of the samples associated to Fig. 2d was measured using an LDH cytotoxicity assay. Shown is the mean of the absorbance values collected from an experiment performed in triplicate +/- SD. ns: non-significant. Statistical significance was determined using a t-test.

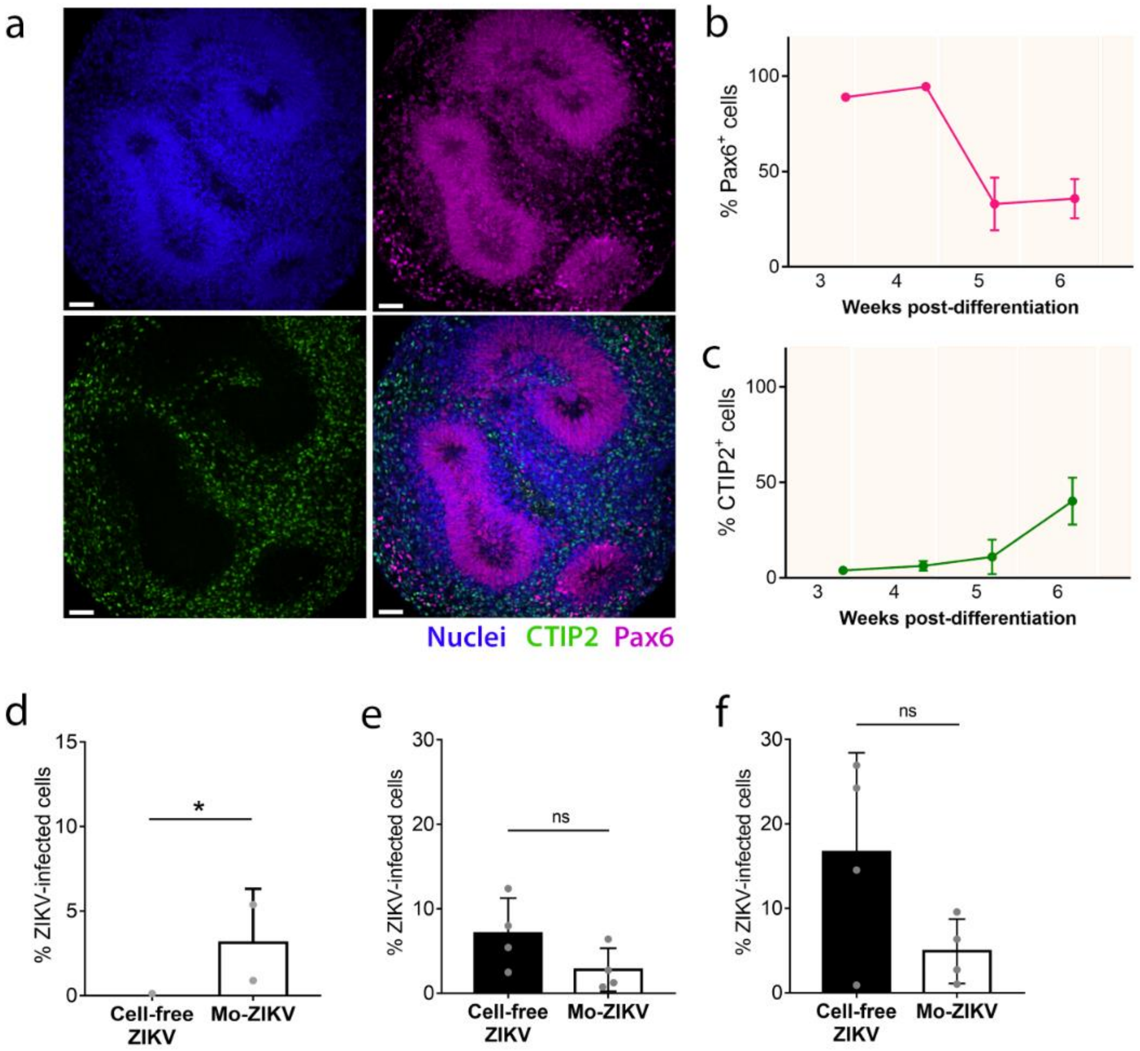

Supplemental Figure 4. Characterization of hESC-derived cerebral organoids. (**a**) At 40 days of differentiation (6 wpd), the organoids were processed for immunofluorescence and stained for CTIP2 and Pax6. Shown are representative pictures acquired on an inverted microscope coupled to the Andor Dragonfly spinning disk. Images were processed using Imaris x64 version 9.2. Scale bar: 50 µm. (**b-c**) At indicated timepoints of culture, the organoids were fixed and processed for flow cytometry using a Pax6 (**b**) and CTIP2 (**c**) staining. The graphs show the percentage of cells positive for either of these two populations. The points in the graph represent the mean +/- SD. (**d-f**) The organoids were co-cultured with cell-free ZIKV and ZIKV-infected monocytes. At 2 dpi (**d**) or 9 dpi (**e-f**), they were fixed, dissociated and analyzed by fax. Shown is the % of ZIKV-infected cells obtained by staining for ZIKV-E with the 4G2 antibody of organoids that were infected at 3 wpd (**e**) and 5 wpd (**d** and **f**). The bar graphs show the mean of two experiments performed in duplicate +/- SD with monocytes from two donors. Two-tailed *p*-value is ns, non-significant or < 0.05 (*). Statistical significance was determined using a t-test.

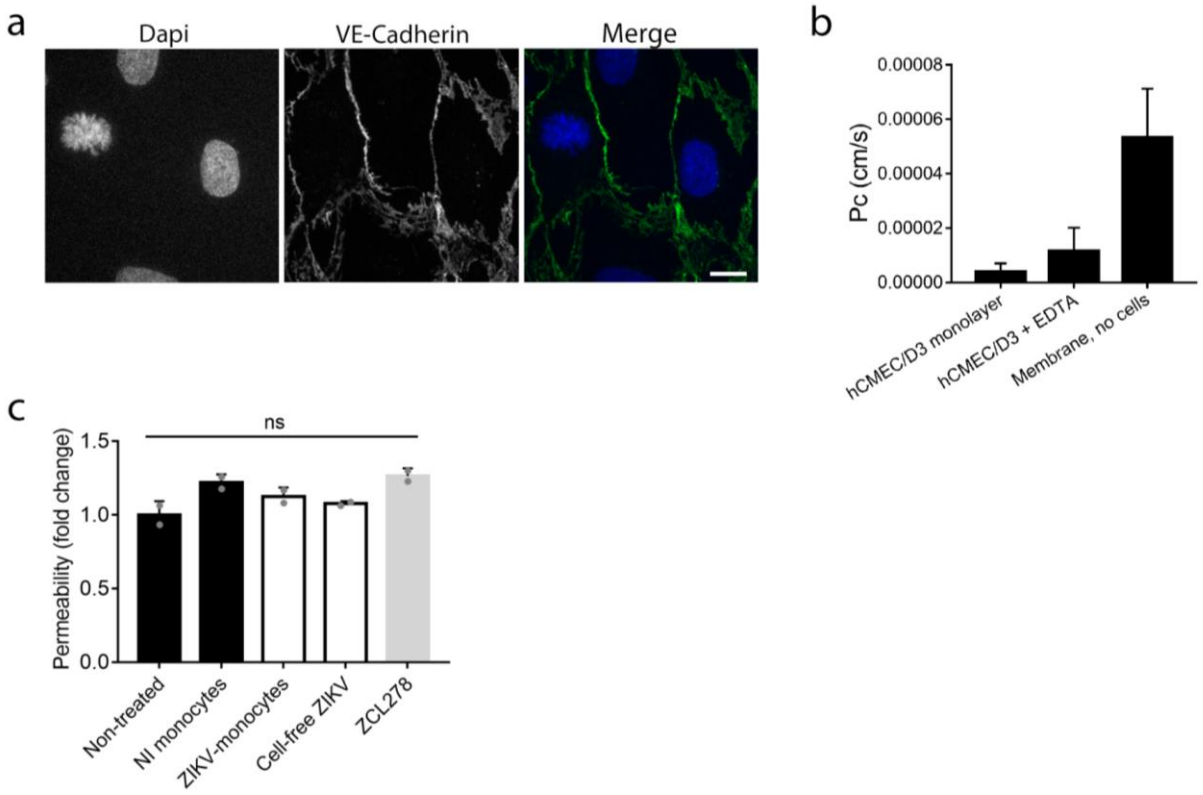

Supplemental Figure 5. Characterization of the BBB endothelial layer formed by hCMEC/D3 cells. (**a**) The hCMEC/D3 cells were seeded on coverslips pre-coated with rat collagen type 1. The cells were cultured for 7 days before the cells were fixed and processed for immunofluorescence. The staining was performed using a goat anti-VE-Cadherin antibody (green) and Dapi (blue) and images were acquired with an epifluorescence microscope. Scale bar: 10 µm. (**b**) A Lucifer yellow permeability assay was performed on a monolayer of hCMEC/D3 cells growing in a transwell insert for 7 days. The cells were incubated with 50 µM Lucifer yellow with or without 5 mM EDTA for 2 h at 37°C. As a control, Lucifer yellow was also added to an insert with no cells (Membrane, no cells). Fluorescence was quantified using a microplate reader. The graph shows permeability coefficient (Pc) values calculated for all conditions using a calibration curve as a reference. The fold-change of the Pc of EDTA-treated cells compared with non-treated cells was significantly greater (FC = 3.39 +/- 1.04, *p*-value = 0.008). (c) A Lucifer Yellow permeability assay was performed under the indicated conditions in order to measure a permeability coefficient according to standard calculations. Shown are the mean +/- SD of the fold change with respect to the non-treated (NT) control.

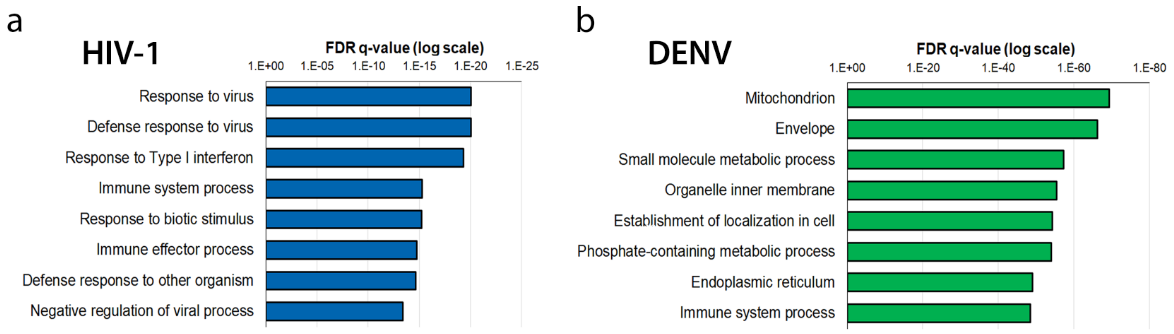

Supplemental Figure 6. Upregulated protein pathways upon HIV-1 or DV2 infection of monocytes. Monocytes isolated from two healthy donors were infected with HIV-1 NL(AD8) or DENV serotype 2 (MOI 1). At 48 hpi, the cells were thoroughly washed and processed for quantitative proteome profiling using liquid chromatography coupled to tandem mass spectrometry (LC-MS/MS). Ontology analysis of the upregulated proteins (virus over mock) were identified using GSEA using 5% FDR for (**a**) HIV-1 (p-value < 0.0005) and 1% FDR for (**b**) DENV (p-value < 0.001).

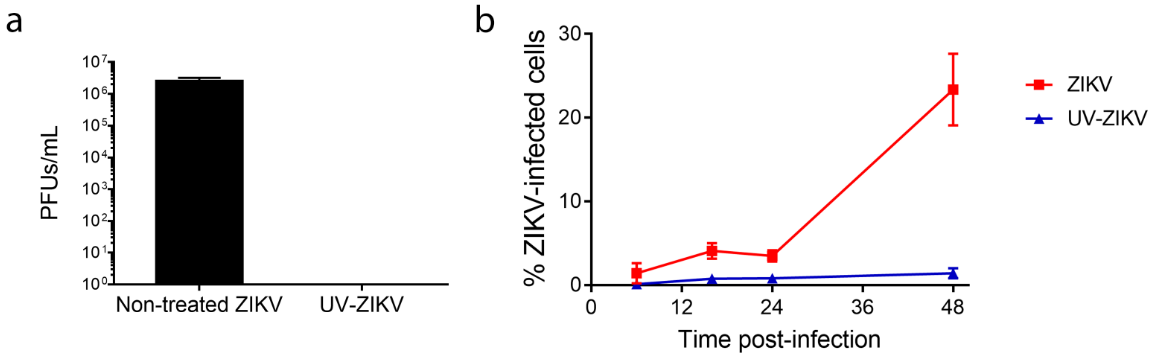

Supplemental Figure 7. Characterization of UV-inactivated ZIKV. (**a**) ZIKV was inactivated using UV radiation. The infectivity of the viral particles was measured by plaque assay on Vero cells. No plaque could be seen in the UV-treated condition even when using undiluted samples. (**b**) Monocytes were infected with untreated virus (red line, squares) at MOI 1 or with equivalent volume of UV-inactivated virus (blue line, triangles) for indicated times. The percentage of ZIKV-infected cells ($NS2B^+$) was measured by flow cytometry.

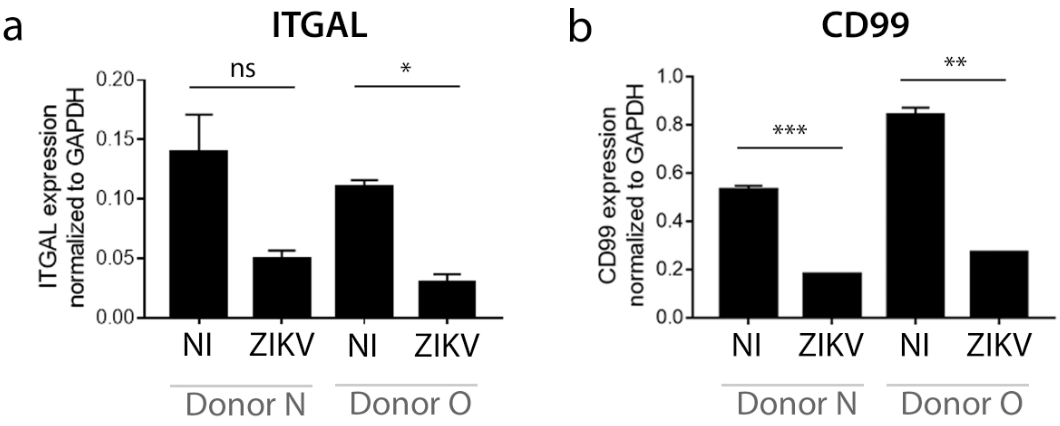

Supplemental Figure 8. ZIKV-infected monocytes exhibit lower mRNA expression levels of adhesion markers. (**a**) Monocytes isolated from two healthy donors were infected with ZIKV (MOI 1). At 48 hpi, total RNA was extracted and RT-qPCR was performed using ITGAL (**a**) and CD99 (**b**) specific primers. The graph represents the mean of a duplicate +/- SD of the expression of the indicated genes normalized to GAPDH. The two-tailed *p*-value is ns, non-significant, < 0.05 (*), 0.005 (**) or 0.0005 (***). Statistical significance was determined using a t-test.

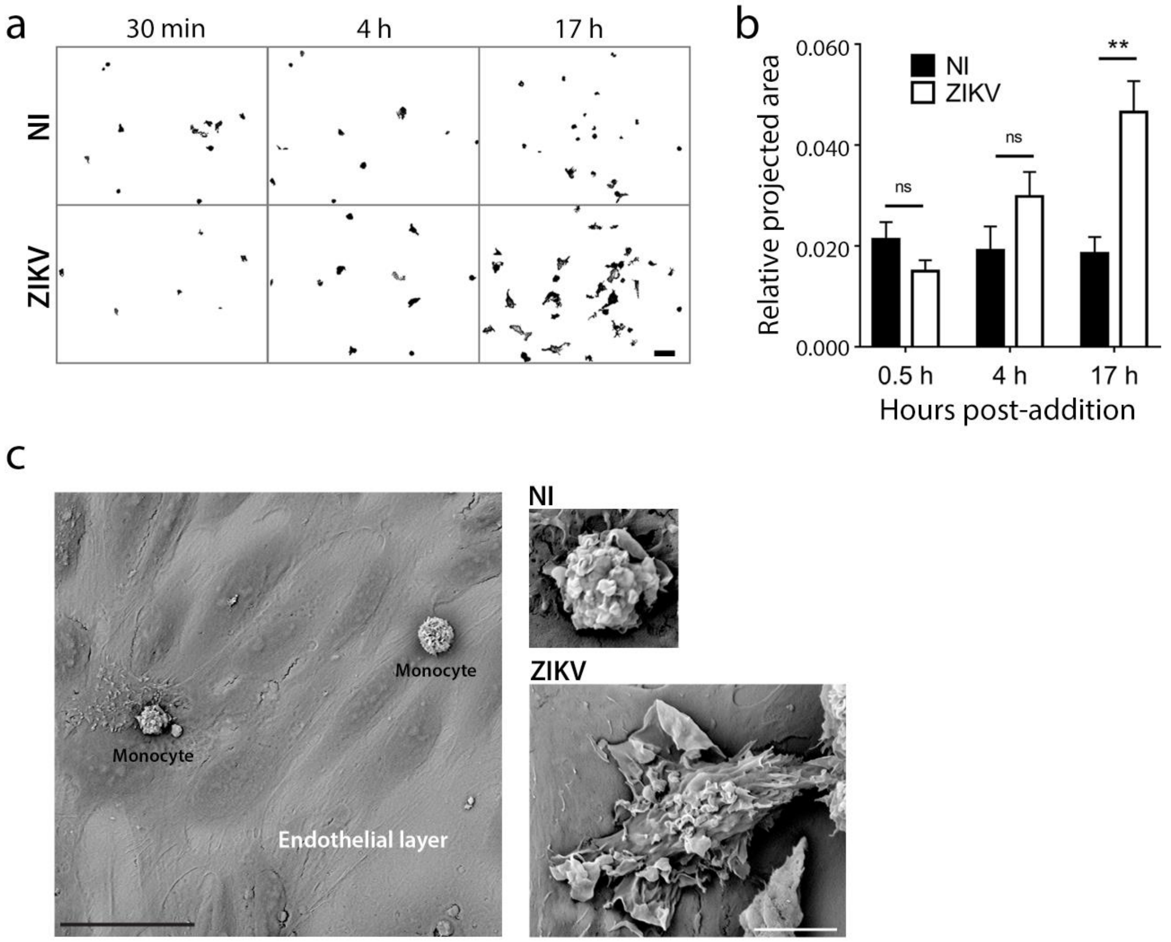

Supplemental Figure 9. ZIKV induces spreading of human primary monocytes. (**a**) ZIKV-infected or non-infected monocytes from two donors were added to hCMEC/D3 cells previously grown on glass coverslips, co-cultured for 0.5 h, 4 h or 17 h, thoroughly washed, fixed, and stained with an anti-CD45 primary antibody. Images were acquired in a spinning disk confocal microscope. Segmentation on the monocyte staining was performed, and a mask was created (black) with ImageJ. Images show 10 representative fields of view. Scale bar: 20 µm. (**b**) Quantification of the Relative projected area was performed as in Fig. 6b. The two-tailed *p*-value is ns, non-significant or < 0.005 (**). Statistical significance was determined using a t-test. (**c**) Non-infected monocytes or monocytes infected for 48 h with ZIKV (MOI 1) were co-cultured with hCMEC/D3 cells on coverslips, and further incubated for 4 h at 37°C. The samples were fixed, processed for scanning electron microscopy (SEM), and imaged with a Phenom ProX Desktop scanning electron microscope. The images show an overview of an endothelial layer with monocytes attached onto it (left panel, Scale bar: 30 µm) and examples of non-infected and ZIKV-infected monocytes (right panels, Scale bar: 5 µm).

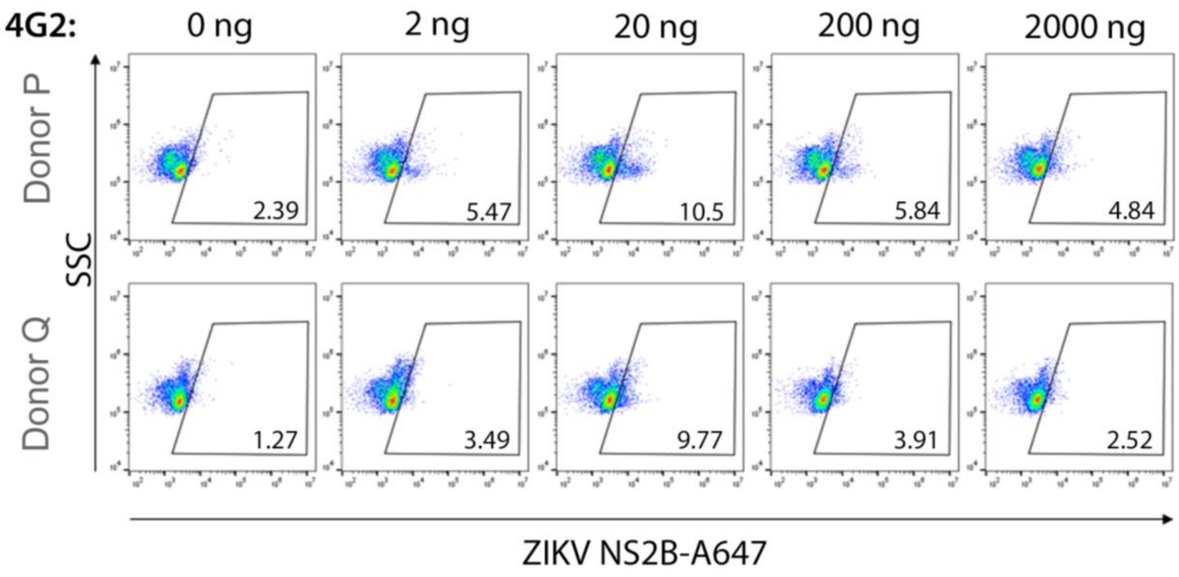

Supplemental Figure 10. Antibody-dependent enhancement (ADE) capability of the 4G2 antibody. Monocytes were infected with ZIKV in the presence of the indicated concentration of the pan-*Flaviviridae* antibody clone 4G2 for 48 h. Cells were fixed, permeabilized and stained using a ZIKV NS2B antibody coupled to an Alexa Fluor 647. The best concentration, used for all ADE-ZIKV infections, was determined at 20 ng/mL.

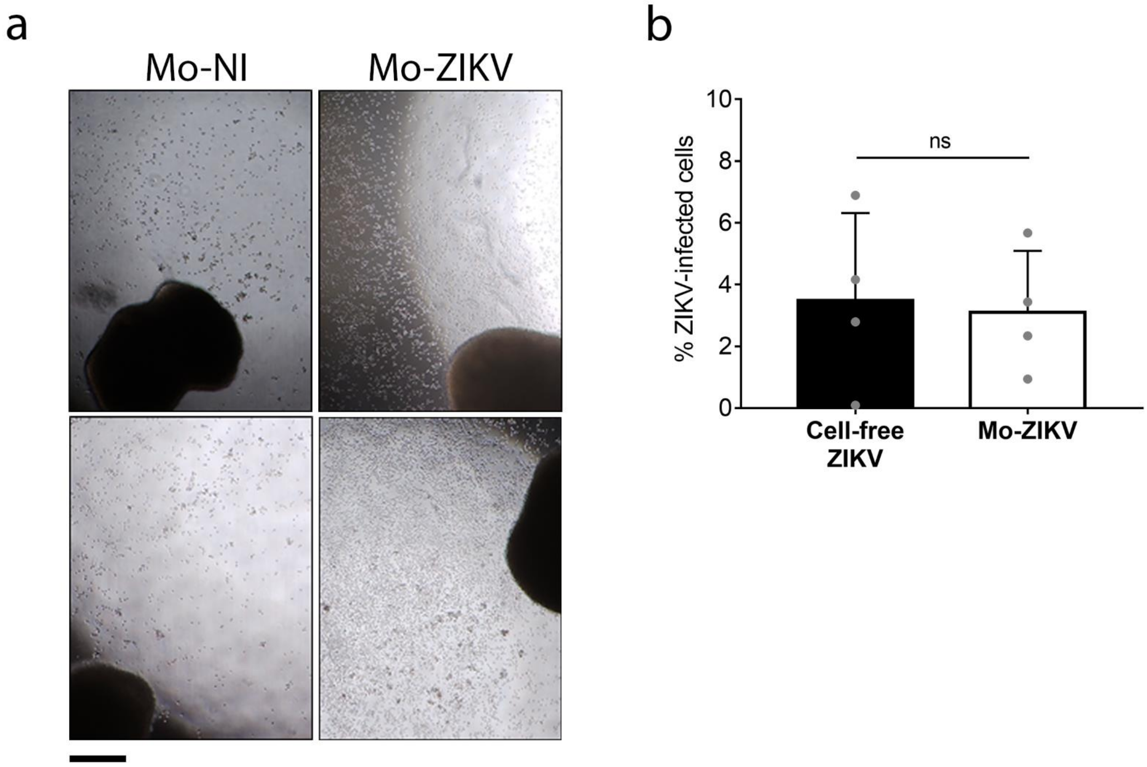

Supplemental Figure 11. Characterization of ZIKV-infected monocytes interactions with cerebral organoids upon transmigration. (**a**) Neural organoids were put under a transwell where hCMEC/D3 cells were previously grown for 7 days. On top of the transwell, either ZIKV-infected monocytes, not-infected monocytes, cell-free ZIKV or mock were added. After an overnight incubation, the transwells were removed. Shown are the images taken of the bottom part of the transwell after this initial overnight incubation. Lower and upper panel correspond to experiments done with two different monocyte donors. Scale bar: 250 µm. (**b**) After further 9 days of incubation, the organoids were fixed and processed for flow cytometry. The percentage of infected cells was obtained by staining for ZIKV-E. The bar graphs represent the mean of an experiment performed in duplicate +/- SD. ns: non-significant.

Supplementary Table 1. Antibodies

Antibody name	Dilution	Vendor	Catalogue number
**Primary, labeled**			
CD3-PB	1:175	Beckman Coulter	A93687
CD4-APC-AF750	1:175	Beckman Coulter	A94682
CD8-AF700	1:175	Beckman Coulter	B76279
CD14-PC7	1:175	Beckman Coulter	A22331
CD16-PE	1:175	Beckman Coulter	A07766
CD19-ECD	1:175	Beckman Coulter	IM2708U
CD45-KO	1:175	Beckman Coulter	B36294
CD56-PC5.5	1:175	Beckman Coulter	B49189
CD45-VioBright515	1:100	Miltenyi Biotech	130-110-778
CD156a-APC	1:20	Miltenyi Biotech	130-104-896
CD62E-APC	1:20	Miltenyi Biotech	130-104-685
CD62L-FITC	1:20	Miltenyi Biotech	130-102-465
CD62P-APC	1:50	Miltenyi Biotech	130-119-157
CD54-APC	1:20	Miltenyi Biotech	130-103-910
CD102-APC	1:50	Miltenyi Biotech	130-114-412
CD106-FITC	1:20	Miltenyi Biotech	130-104-162
CD31-FITC	1:50	Miltenyi Biotech	130-110-806
CD146-FITC	1:20	Miltenyi Biotech	130-097-934
CD11a-FITC	1:20	Miltenyi Biotech	130-105-478
CD11b-APC-Vio770	1:50	Miltenyi Biotech	130-110-556
CD11c-FITC	1:20	Miltenyi Biotech	130-114-105
CD49d-FITC	1:20	Miltenyi Biotech	130-099-720
CD29-FITC	1:20	Miltenyi Biotech	130-101-255
CD18-FITC	1:20	Miltenyi Biotech	130-101-242
CD43-FITC	1:50	Miltenyi Biotech	130-114-749
CD47-FITC	1:20	Miltenyi Biotech	130-101-345
CD162-FITC	1:20	Miltenyi Biotech	130-104-753
CD169-APC	1:20	Miltenyi Biotech	130-098-645
CD209-FITC	1:20	Miltenyi Biotech	130-109-647
CD99-FITC	1:20	Miltenyi Biotech	130-104-363
CCR2-PE Cy7	1:20	Miltenyi Biotech	130-103-901
CD144-AF647	1:20	BD Biosciences	561567
**Primary, non-labeled**			
NS2B	1:150	GeneTex	GTX133308
NS1	1:150	GeneTex	GTX133307
Rabbit anti-Flavivirus group antigen [D1-4G2-4-15 (4G2)]	1:150	Absolute Antibody	Ab00230-23.0
Mouse anti-Flavivirus group antigen [D1-4G2-4-15 (4G2)]	1:150	Novus Biologicals	NBP2-52709
NS1 clone 3C2	1:500	CDC Immuno-Diagnostic Development Team, USA	
CD68	1:100	Leica	NCL-L-CD68
CD163	1:50	Leica	NCL-L-CD163
CD14	1:100	Abcam	ab183322
CD45	1:100	Leica	NCL-L-LCA
Calbindin- D-28K	1:1500	Merck	SAB4200543

VE-Cadherin	1:300	R&D Systems	AF938-SP
CTIP2 (Bcl11b)	1:500	BioLegend	650603
Pax6	1:500	BioLegend	901302
**Secondary, labeled**			
Donkey anti-mouse AF488	1:1000	Invitrogen	R37114
Donkey anti-goat AF488	1:500	Invitrogen	A11055
Donkey anti-rabbit AF568	1:500	Invitrogen	A10042
Donkey anti-rat AF488	1:500	Invitrogen	A21208
Donkey anti-mouse AF647	1:500	Invitrogen	A31571
