# Supplementary material for: Zika virus enhances monocyte adhesion and transmigration favoring viral dissemination to neural cells
Source: Nat Commun. 2019 Sep 27;10:4430. doi: 10.1038/s41467-019-12408-x (PMC6764950; doi:10.1038/s41467-019-12408-x)
Supplement: Supplementary file 2 — Description of Additional Supplementary Files [file 41467_2019_12408_MOESM2_ESM.docx]

**Description of Additional Supplementary Files**

**File Name: Supplementary Movie 1**

**Description:** Representative 3D confocal image of the monocytes’ distribution within the vasculature of a zebrafish embryo. Imaging was performed at 6 h post-injection. The endothelial cells of the Tg(fli1a:eGFP) zebrafish embryo are shown in green. The fluorescently labeled human primary monocytes are shown in magenta.

**File Name: Supplementary Movie 2**

**Description:** Live cell imaging of human monocytes moving in the zebrafish vasculature just after injection. The endothelial cells of the Tg(fli1a:eGFP) zebrafish embryo are shown in green. The fluorescently labeled human primary monocytes are shown in magenta. Time stamp is in the format mm:ss.

**File Name: Supplementary Data 1**

**Description:** *List of the proteins modulated upon ZIKV infection*. Upon ZIKV infection, proteome profiling was performed using LC-MS/MS. The table includes the list of proteins differentially expressed between mock and ZIKV-exposed monocytes.
