# Supplementary material for: Zika virus enhances monocyte adhesion and transmigration favoring viral dissemination to neural cells
Source: Nat Commun. 2019 Sep 27;10:4430. doi: 10.1038/s41467-019-12408-x (PMC6764950; doi:10.1038/s41467-019-12408-x)
Supplement: Supplementary file 6 — Reporting Summary [file 41467_2019_12408_MOESM6_ESM.pdf]

nature research

Corresponding author(s): *Double-blind peer review submissions: write DBPR and your manuscript number here instead of author names.*

Last updated by author(s): *YYYY-MM-DD*

Reporting Summary

Nature Research wishes to improve the reproducibility of the work that we publish. This form provides structure for consistency and transparency in reporting. For further information on Nature Research policies, see Authors & Referees and the Editorial Policy Checklist.

Statistics

For all statistical analyses, confirm that the following items are present in the figure legend, table legend, main text, or Methods section.

n/a	Confirmed	
☐	☒	The exact sample size (*n*) for each experimental group/condition, given as a discrete number and unit of measurement
☐	☒	A statement on whether measurements were taken from distinct samples or whether the same sample was measured repeatedly
☐	☒	The statistical test(s) used AND whether they are one- or two-sided<br>*Only common tests should be described solely by name; describe more complex techniques in the Methods section.*
☒	☐	A description of all covariates tested
☒	☐	A description of any assumptions or corrections, such as tests of normality and adjustment for multiple comparisons
☐	☒	A full description of the statistical parameters including central tendency (e.g. means) or other basic estimates (e.g. regression coefficient) AND variation (e.g. standard deviation) or associated estimates of uncertainty (e.g. confidence intervals)
☐	☒	For null hypothesis testing, the test statistic (e.g. *F*, *t*, *r*) with confidence intervals, effect sizes, degrees of freedom and *P* value noted<br>*Give P values as exact values whenever suitable.*
☒	☐	For Bayesian analysis, information on the choice of priors and Markov chain Monte Carlo settings
☒	☐	For hierarchical and complex designs, identification of the appropriate level for tests and full reporting of outcomes
☒	☐	Estimates of effect sizes (e.g. Cohen's *d*, Pearson's *r*), indicating how they were calculated

Our web collection on statistics for biologists contains articles on many of the points above.

Software and code

Policy information about availability of computer code

Data collection	No software was used.
Data analysis	ImageJ for image analysis, Bitplane Imaris (x64 9.2.0) for image analysis, FlowJo (v10.4.2) for analysis of flow cytometry data.

For manuscripts utilizing custom algorithms or software that are central to the research but not yet described in published literature, software must be made available to editors/reviewers. We strongly encourage code deposition in a community repository (e.g. GitHub). See the Nature Research guidelines for submitting code & software for further information.

Data

Policy information about availability of data

All manuscripts must include a data availability statement. This statement should provide the following information, where applicable:

- Accession codes, unique identifiers, or web links for publicly available datasets
- A list of figures that have associated raw data
- A description of any restrictions on data availability

The source data underlying Figures 2-9 (2a, 2b, 2d, 3b, 3e, 4b, 4c, 4e, 5b, 5c, 5d, 5e, 5f, 5h, 6b, 6c, 6d, 7e, 8b, 8e, 8g, 9c) are provided as a Source Data file. The complete dataset resulting from the mass spectrometry proteomics analysis has been deposited to the ProteomeXchange Consortium via the PRIDE partner repository with the dataset identifier PXD014002. All other data are available from the corresponding author upon reasonable request.

Field-specific reporting

Please select the one below that is the best fit for your research. If you are not sure, read the appropriate sections before making your selection.

☒ Life sciences ☐ Behavioural & social sciences ☐ Ecological, evolutionary & environmental sciences

For a reference copy of the document with all sections, see nature.com/documents/nr-reporting-summary-flat.pdf

Life sciences study design

All studies must disclose on these points even when the disclosure is negative.

Sample size	No sample-size calculations were performed. Sample size was determined to be adequate based on the magnitude and consistency of measurable differences between groups.
Data exclusions	On principle, data were only excluded for failed experiments, such as negative results on our positive control. Moreover, the monocytes from donors that were pre-activated upon isolation were excluded from the experiments.<br>No monocyte infection in one donor in figure 2b and in one replicate in fig 4c was detected for unknown reason. the data were excluded from analyses. This is mentioned in the text and the data are provided in the Source Data file associated to the manuscript.
Replication	Experiments were replicated multiple times with reproducible results indicated in the figure legends.<br>When donor-dependent variability was observed, the data were reported individually for each donors (Figure 2b and 2d) and discussed in the text.<br>We failed at infecting human primary monocytes in two experiments (fig 2c one donor and fig 4c one replicate) for unknown reason. These data were excluded (see "Data exclusion" above and "Source Data file"). Besides these two cases, all the data were fully reproducible as presented in the figures.
Randomization	For fish experiments, one fish was imaged for each condition before the next replicate was imaged.
Blinding	The organotypic cerebellar slices were analyzed blind. The quantitative data reported are not subjective but rather based on automated analyses.

Reporting for specific materials, systems and methods

We require information from authors about some types of materials, experimental systems and methods used in many studies. Here, indicate whether each material, system or method listed is relevant to your study. If you are not sure if a list item applies to your research, read the appropriate section before selecting a response.

Materials & experimental systems

n/a	Involved in the study	
☐	☒	Antibodies
☐	☒	Eukaryotic cell lines
☒	☐	Palaeontology
☐	☒	Animals and other organisms
☐	☒	Human research participants
☒	☐	Clinical data

Methods

n/a	Involved in the study	
☒	☐	ChIP-seq
☐	☒	Flow cytometry
☒	☐	MRI-based neuroimaging

Antibodies

Antibodies used	A table with a list of all antibodies used, its dilutions, provider, and catalogue number was included as Supplementary Table 2.
Validation	A table with a list of all antibodies used, its dilutions, provider, and catalogue number was included as Supplementary Table 2.

Eukaryotic cell lines

Policy information about cell lines

Cell line source(s)	The human Cerebellar Microvascular Endothelial Cells D3 (hCMEC/D3) cells were provided by S. Bourdoulous, Institut Cochin, France. Vero cells were obtained from the ATCC (CCL-81). C6/36 cells were provided by M. Flamand, Institut Pasteur, France.
Authentication	Expression of different endothelial proteins (like VE-Cadherin and Occludin) was assessed in the hCMEC/D3 using immunofluorescence. Besides, permeability of the monolayer was measured by TEER and Lucifer Yellow (see Materials and Methods).

Mycoplasma contamination	All cell lines were tested with the MycoAlertTM Mycoplasma Detection Kit from Lonza.
Commonly misidentified lines (See ICLAC register)	No misidentified lines were used.

Animals and other organisms

Policy information about studies involving animals; ARRIVE guidelines recommended for reporting animal research

Laboratory animals	Tg(fli1a:eGFP) Zebrafish (Danio rerio) embryos<br>CD1 mice
Wild animals	The study did not involve wild animals.
Field-collected samples	The study did not involve samples collected from the field.
Ethics oversight	Zebrafish: All animal procedures were performed in accordance with French and European Union animal welfare guidelines.<br>Mice: All animal procedures were performed in accordance with French and European Union animal welfare guidelines.<br><br>Animal experiments were all approved in advance by the Comité Régional d'Ethique en Matière d'Expérimentation Animale de Strasbourg (CREMEAS) under regulation CEEA35 approved by the French Ministère de l'enseignement supérieur et de la recherche.

Note that full information on the approval of the study protocol must also be provided in the manuscript.

Human research participants

Policy information about studies involving human research participants

Population characteristics	Human blood donors for experiments were anonymous.
Recruitment	Human blood donors for experiments were anonymous.
Ethics oversight	In case of the fetus tissue: both the initial receipt of human samples [061116SZ] and the secondary use [102517GH] were submitted for human subjects review in accordance with standard NCEZID procedures, and both were determined to be outside the scope of IRB review requirements under 45 CFR 46 [pre-2018 rule] by the NCEZID Senior Human Subjects Advisor, as authorized by CDC institutional policy, as the fetuses are not living individuals [46 CFR 46.102(d), pre-2018 rule].

Note that full information on the approval of the study protocol must also be provided in the manuscript.

Flow Cytometry

Plots

Confirm that:

- [x] The axis labels state the marker and fluorochrome used (e.g. CD4-FITC).
- [x] The axis scales are clearly visible. Include numbers along axes only for bottom left plot of group (a 'group' is an analysis of identical markers).
- [x] All plots are contour plots with outliers or pseudocolor plots.
- [x] A numerical value for number of cells or percentage (with statistics) is provided.

Methodology

Sample preparation	Sample preparation listed in Methods.
Instrument	Navios (Beckman Coulter), LSR II (BD Biosciences), MACS Quant (Miltenyi Biotec), Cytoflex (Beckman Coulter), and Novocyte (ACEA Biosciences)
Software	FlowJo version v10.4.2
Cell population abundance	The monocytes were around 10% of total PBMCs. Purity of the population was assessed with a CD14 antibody.
Gating strategy	To determine the proportion of ZIKV infected cells, the samples were stained with NS2B or 4G2 antibodies. Gating strategy: 1) in FSC/SSC gate for living cells, 2) using the non-infected control define the gate for NS2b or 4G2+ cells, 3) apply this gate to all samples.

- [ ] Tick this box to confirm that a figure exemplifying the gating strategy is provided in the Supplementary Information.
